# Ecophysiological Suitability of *Batrachochytrium dendrobatidis* in Mexico

**DOI:** 10.1007/s10393-025-01734-w

**Published:** 2025-07-15

**Authors:** Aldo Gómez-Benitez, Erika Adriana Reyes-Velázquez, Karla Pelz-Serrano, Laura Heredia-Bobadilla, Armando Sunny-García, Víctor Daniel Ávila-Akerberg

**Affiliations:** 1 Departamento de Ciencias Ambientales, División de Ciencias Biológicas y de La Salud, Universidad Autónoma Metropolitana Unidad Lerma, Avenida de Las Garzas #10 El Panteón, C.P. 52005 Lerma de Villada Estado de México, Mexico; 2 Red de Investigación y Divulgación de Anfibios y Reptiles MX, Toluca de Lerdo, Estado de México Mexico; 3 Centro de Investigación en Ciencias Biológicas Aplicadas, Facultad de Ciencias, Universidad Autónoma del Estado de México, Instituto Literario 100, Colonia Centro, C.P. 50000 Toluca de Lerdo, Estado de México Mexico; 4 Consejo Mexiquense de Ciencia y Tecnología, Paseo Cristóbal Colón #112ª Residencial Colón y Colonia Ciprés, C.P. 50120 Toluca de Lerdo, Estado de México Mexico

**Keywords:** chytrimiomycosis, endemic amphibians, mechanistic model, natural protected areas, physiological constraints, reproduction

## Abstract

*Batrachochytrium dendrobatidis* (*Bd*) is a fungal pathogen responsible for amphibian population declines worldwide. In Mexico, understanding the potential distribution of *Bd* is crucial for conservation. Here, we developed an ecophysiological suitability index that integrates five key physiological parameters related to temperature. The environmental variables from WorldClim v2.1 were used to derive spatial representations of *Bd’s* thermal responses. A multicriteria evaluation combined these parameters into an ecophysiological suitability index. This index was projected to 2050 and 2070 under two contrasting climate change scenarios. We also analyzed the overlap between *Bd’s* suitability, natural protected areas (NPAs), and endemic amphibian geographic distributions to assess conservation implications. Our results indicate that *Bd* suitability is highest in the mountainous regions of central Mexico and declines significantly in coastal and lowland areas. By 2050 and 2070, both climate change scenarios predict reduced suitability across most of Mexico. Thirty-five percent of federal NPAs and 53% of state NPAs had high suitability for *Bd*. Endemic amphibians inhabiting mountains face greater suitability for *Bd* in their habitats. Our findings highlight the utility of mechanistic models for predicting pathogen distributions under environmental constraints. This approach provides actionable insights for prioritizing conservation efforts, including habitat management and amphibian population monitoring.

**Supplementary Information:**

The online version contains supplementary material available at 10.1007/s10393-025-01734-w.

## Introduction

### History and Spread of *Batrachochytrium dendrobatidis* Around the World

The chytrid fungus *Batrachochytrium dendrobatidis* (*Bd*), which is responsible for the disease chytridiomycosis in amphibians, was first described and named by Longcore et al. (1999) in the late 90 s; this is the first study to identify *Bd* as the causative agent of amphibian mortality in both captive and wild populations in North America, Central America and Australia. However, retrospective studies suggest that *Bd* has been causing rapid population declines since the 1970s, particularly in Australia and Central America (Berger et al., 2016). Globally, *Bd* has affected more than 500 amphibian species, causing a decline of up to 200 species, and is presumed to have driven at least 90 amphibian populations to local extinction (Scheele et al., 2019; Skerratt et al., 2007). Historical evidence places the presence of *Bd* as early as 1888 in the USA and 1894 in Mexico (Basanta et al., 2020; Talley et al., 2015).

The origin of *Bd* has been the subject of ongoing debate, with multiple proposed sources (Goka et al., 2009; James et al., 2009; Weldon et al., 2004). East Asia, particularly the Korean peninsula, exhibits the highest genetic diversity and is hypothesized to be the ancestral origin of *Bd* (O’Hanlon et al., 2018). From this geographic origin, hypervirulent *Bd* variants have spread globally, largely driven by human trade during the twentieth century (Fu and Waldman, 2022; O’Hanlon et al., 2018). Legal pathways include the trade of highly commercialized amphibian species which could act as reservoirs of the pathogen (Schloegel et al., 2009; Vredenburg et al., 2013). Conversely, illegal trade poses a major risk for *Bd* dispersion and has been well documented in the trade of several species in Mexico (García-Feria et al., 2017). The *Batrachochytrium dendrobatidis* cryptic early arrival to Mexico and ongoing illegal trade underscore the urgent need to understand its distribution under local climatic conditions and the risk for its dispersal.

### Effects of *Bd* Infection on Amphibians and the *Bd* Life Cycle

Chytridiomycosis is a lethal skin disease that disrupts essential physiological processes in amphibians, such as respiration and water balance, both of which are regulated through the skin (Fisher et al., 2009; Voyles et al., 2011). *Batrachochytrium dendrobatidis* colonizes keratinized tissues, causing hyperkeratosis in the epidermal layer of adult amphibians, while in tadpoles, it primarily affects the buccal apparatus and is generally nonlethal (Fellers et al., 2001; Van Rooij et al., 2015). Additionally, infected amphibians may exhibit behavioral changes, including reduced feeding and lethargy, which further promote their decline (DeMarchi et al., 2015; Hanlon et al., 2015).

**Figure 1:**
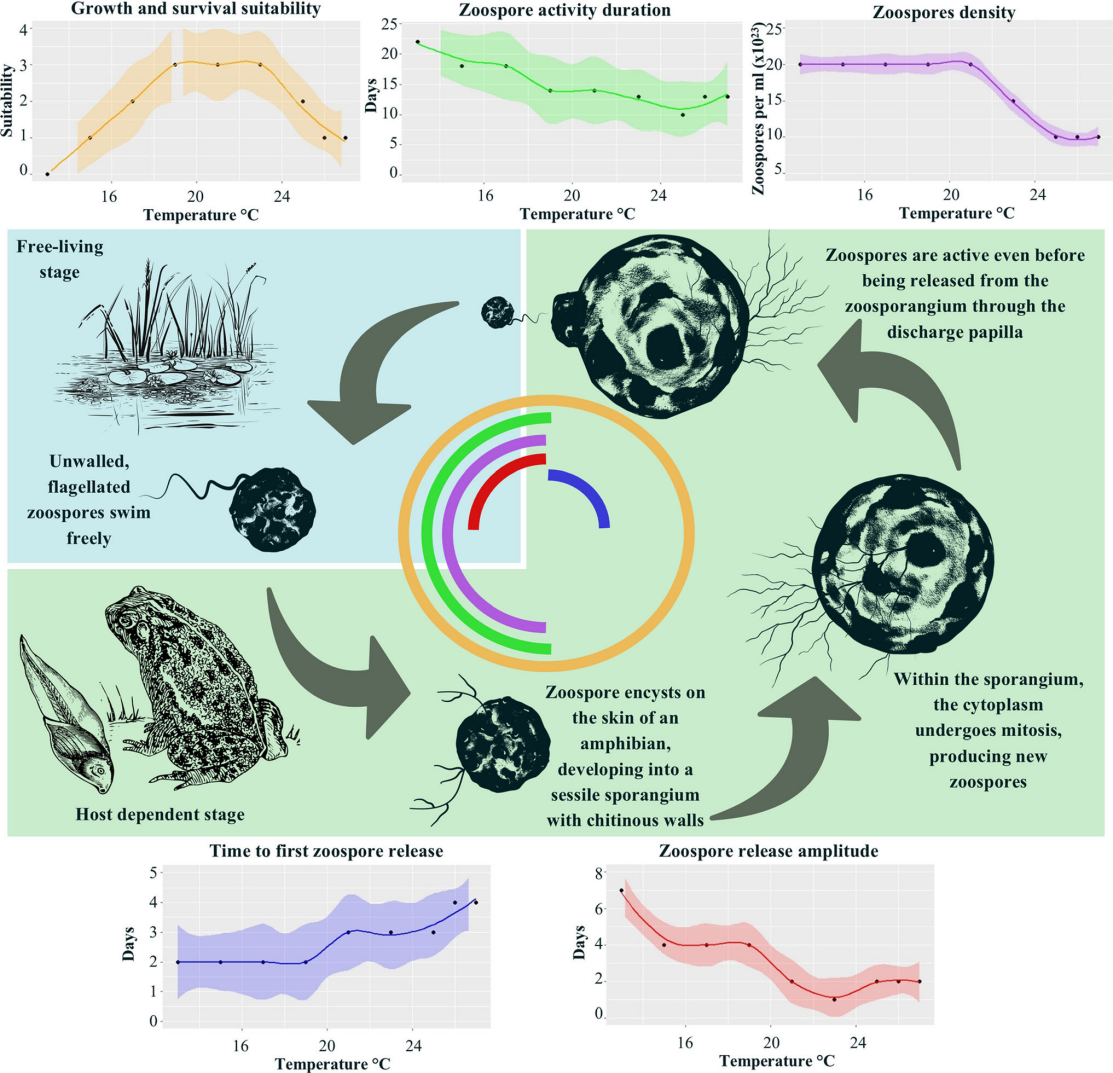
Physiological parameters of *Batrachochytrium dendrobatidis* in relation to temperature and their relevance during different stages of the life cycle. The graphs above and below the central life cycle diagram show the temperature dependence of growth suitability, duration of zoospore activity, zoospore density, time to first zoospore release, and zoospore release amplitude based on data from Piotrowski et al. (2004), Stevenson et al. (2013), and Voyles et al. (2017). The colored arcs in the central diagram highlight the stages of the *Bd* life cycle where these parameters are most critical, and the colors correspond to the characteristic graphs they represent, adapted from Berger et al. (2005) and Longcore et al. (1999). The weights assigned to each parameter (growth suitability: 0.4; zoospore density and activity duration: 0.2 each; time to first zoospore release and zoospore release amplitude: 0.1 each) reflect their respective roles in *Bd* persistence and transmission.

*Batrachochytrium dendrobatidis* undergoes two main stages in its life cycle: a motile (dispersion) stage and a stationary stage (Berger et al., 2005). In the motile stage, unwalled, flagellated zoospores swim freely in search of a suitable host (Berger et al., 2005; Longcore et al., 1999). Once a zoospore encysts on the skin of an amphibian, it transitions to the stationary stage, during which it develops into a sessile sporangium with chitinous walls (Berger et al., 2005; Longcore et al., 1999). Within the sporangium, the cytoplasm undergoes mitosis, where new zoospores are produced that are active even before being released from the zoosporangium (Berger et al., 2005; Longcore et al., 1999). The life cycle and natural history traits of *Bd* are strongly influenced by environmental conditions, particularly, temperature have a critical role on the development and dispersal of *Bd* (Voyles et al., 2012; Fig. 1). *Batrachochytrium dendrobatidis* thrives within a temperature range of 4–25 °C, with optimal growth observed at 17–25 °C and temperatures above 30 °C are lethal to zoospores (Piotrowski et al., 2004). Under low-temperature conditions, *Bd* slows its growth and maturation, instead prioritizing zoospore activity, zoospore density, fecundity, and infectivity, demonstrating the remarkable ability of *Bd* to employ trade-offs to adapt to unfavorable thermal conditions (Stevenson et al., 2013; Voyles et al., 2012; Woodhams et al., 2008).

### *Batrachochytrium dendrobatidis* in Mexico

Mexico is not exempt from the global issue caused by *Bd*, as according to reports of 78 affected species across 14 of the country’s 32 states, the mountainous regions of the nation are identified as the areas with the highest recorded presence of the pathogen, particularly in cloud forests (Bolom-Huet et al., 2019). The oldest records in the country correspond to *Anaxyrus punctatus* and *Hyliola cadaverine* (*Pseudacri*s *cadaverine*; Faivovich et al., 2018) in the Baja California Peninsula in 1894 (Basanta et al., 2020). Nevertheless, the chytrid fungus was not officially registered in Mexico until 2005; it was a register of *Rana tarahumarae* collected from the 1980s to 2000s the first register of *Bd* in national territory (Hale et al., 2005). The impacts of *Bd* in Mexico have been well documented. Cheng et al. (2011) reported that the mass extirpation of Plethodontidae species previously reported by Rovito et al. (2009) in southern Mexico was related to *Bd* emergence and high prevalence. Recently, Basanta et al. (2021) reported the occurrence of *Bd* in two dead amphibian species in central Mexico, *Ambystoma altamirani* and *Rana montezumae*, suggesting that the presence of the fungus is related to the death of the individuals. The most affected regions in the country, or at least the regions with more records of *Bd*, are the Trans-Mexican Volcanic Belt, the Sierra Madre del Sur and the mountain ranges in Chiapas, mainly in environments with natural vegetation (Bolom-Huet et al., 2019). Additionally, Natural Protected Areas (NPAs) in Mexico—legally defined as zones that conserve representative ecosystems and their ecological functions (SEMARNAT, 1988)—harbor a substantial proportion of the country’s endemic amphibian diversity (up to 60% in federal NPAs and 35% in private reserves; Quintero-Vallejo and Ochoa-Ochoa, 2022). Several of these NPAs have already confirmed *Bd* presence, undermining their conservation objectives; for example, Monarch Butterfly Biosphere Reserve (Nava-González et al., 2010), El Vizcaíno Biosphere Reserve, Sierra La Laguna Biosphere Reserve (Luja et al., 2012), and Iztaccíhuatl–Popocatépetl National Park (Van Rooij et al., 2011). This overlap of high amphibian endemism and *Bd* suitability underscores the urgent need to quantify *Bd* risk within NPAs and to develop targeted strategies for prevention and mitigation.

### Distribution Models of Pathogens

Species distribution models (SDMs) are valuable tools for understanding ecological and biogeographical patterns; however, their application to pathogen distributions presents significant limitations, particularly when predicting future spread (Murray et al., 2011a, b). A major challenge in modeling pathogen distributions lies in the strong dependence between hosts, vectors, and pathogens since incomplete or sparse data on host or vector distributions can lead to inaccurate predictions (Mowry et al., 2024). This issue is exacerbated when attempting to project future scenarios or anticipate new invasion fronts, as SDMs must be robust enough to extrapolate meaningfully or, alternatively, should minimize extrapolation to avoid unreliable predictions (Rohr et al., 2011).

One approach to mitigate these limitations is the use of mechanistic or process-based models, which integrate the physiological and ecological constraints of the pathogen itself rather than relying solely on host distribution patterns (Kearney and Porter, 2009; Rose Vineer, 2020). Mechanistic models integrate physiological tolerances, behavioral traits, or morphological characteristics directly into SDMs, treating organisms not merely as occurrence points on a map, as is typical in correlative models, but as entities with measurable responses to environmental variables (Kearney and Porter, 2009). Unlike correlative SDMs, which infer species–environment relationships from occurrence data and associated environmental predictors, mechanistic models explicitly simulate the biological processes underlying development, survival, and reproduction (Buckley et al., 2010; Dormann et al., 2012). This explicit nature requires a more detailed understanding of species’ ecophysiology, and often depends on experimentally derived data, making mechanistic models more data-intensive but potentially more robust under novel environmental conditions or future climate scenarios (Dormann et al., 2012). While mechanistic models do not rely on occurrence data to build projections, such data remains essential for calibration and validation purposes (Dormann et al., 2012; Rose Vineer et al., 2020). These approaches are particularly useful when modeling species or pathogens with well-known environmental constraints, as in the case of *Bd*, whose life cycle and temperature-dependent traits make it more suitable for mechanistic modeling.

Mechanistic approach has proven particularly effective for modeling the distribution of *Bd*, as demonstrated by Murray and Skerratt (2012). Unlike correlative models, which assume that species are in equilibrium with their environment—meaning they occur in all suitable habitats—pathogens often violate this assumption, necessitating the incorporation of true-absence data to improve predictive power (Václavík and Meentemeyer, 2009). Moreover, dispersal constraints are frequently underestimated in invasive pathogen modeling (Václavík and Meentemeyer, 2009). In the specific case of *Bd*, where human activity serves as the primary vector for dispersal (Skerratt et al., 2007) and human population density is a known predictor of *Bd* presence (Murray et al., 2011a, b), correlative SDMs could fail to account for the true drivers of pathogen persistence. Rather than assuming dispersal limitations, it is more appropriate to consider that *Bd* could reach any location accessible to humans, while habitat suitability determines the likelihood of establishment and proliferation.

Given these considerations, we propose an ecophysiological suitability index for *Bd* in Mexico based solely on physiological constraints and their relationship with temperature. This method avoids the biases inherent to correlative models, which often overfit predictions to host distributions, and circumvents the limitations imposed by incomplete occurrence data. Additionally, we forecast *Bd’s* future suitability for 2050 and 2070 under two climate change scenarios (SSP370 and SSP585). Finally, we assessed the potential risk of *Bd* within Mexico’s NPAs and across the distributions of 273 endemic amphibian species.

## Methods

### Review of Available Information About *Bd* Ecophysiology

To compile information on the physiological limitations of *Bd* related to temperature, a systematic literature search was conducted using Google Scholar, Scopus, and ResearchGate. Keywords included *Batrachochytrium dendrobatidis* temperature tolerance, *Bd* thermal performance, chytridiomycosis physiological constraints, and *Bd* growth and reproduction. We prioritized peer-reviewed studies detailing the thermal limits and reproductive biology of the pathogen under varying temperature conditions. Based on these findings, five key ecophysiological parameters were selected for model construction due to their relevance to *Bd* persistence and disease development in amphibians. Selection was guided by the premise that *Bd’s* reproductive biology plays a critical role in disease progression, particularly its ability to persist and develop under suboptimal thermal conditions. At low temperatures, *Bd* exhibits survival strategies that include prolonged zoospore viability, extended thallus maturation times, and increased zoospore production per sporangium (Woodhams et al., 2008). The selected parameters, drawn from Piotrowski et al. (2004), Stevenson et al. (2013) and Voyles et al. (2017), included the following:

- Time to first zoospore release, which varies with temperature: 2 days at 13–19 °C, 3 days at 21–25 °C, and 4–6 days at 26–27 °C.
- The time to maximum zoospore release ranged from 7–9 days at 13 °C, 6 days at 15 °C, 5–6 days at 17 °C, 4–6 days at 19 °C, 4–5 days at 21 °C, 4 days at 24 °C, 4–5 days at 25 °C, 5–6 days at 26 °C, and 6 days at 27 °C.
- Zoospore activity duration was 15–22 days at 13 °C, 13–18 days at 15 °C, 12–18 days at 17 °C, 12–14 days at 19 °C, 11–14 days at 21 °C, 10–13 days at 23 °C, 10 days at 25 °C, 8–13 days at 26 °C, and 9–13 days at 27 °C.
- Zoospore density, which is maximal between 13 and 21 °C, decreases at 23 °C and reaches minimal levels at 25–27 °C.
- Growth and survival suitability were assessed, with an observed growth range of 4–25 °C, optimal growth between 17–25 °C, and peak growth at 22.1–24.6 °C, while zoospore mortality occurred at temperatures exceeding 30 °C.

To refine these parameters and ensure model continuity, intermediate values were estimated using a regression model that best fit the available data. This approach allowed for interpolations across temperature ranges where empirical data were sparse, providing a more comprehensive representation of *Bd’s* thermal response curve (Fig. 1). Additionally, we calculated the zoospore release amplitude, defined as the difference between the time to maximum zoospore release and the time to first zoospore release. This variable provides a more biologically meaningful representation of the duration over which *Bd* actively releases infective zoospores, integrating both reproductive timing and environmental constraints. Given its ecological relevance, the zoospore release amplitude replaced the time to maximum zoospore release in subsequent analyses.

### Determination of the Ecophysiological Suitability Index

To construct an ecophysiological suitability index for *Bd*, we integrated key physiological constraints related to temperature into a spatially explicit framework. The process involved (1) converting physiological parameters into rasterized environmental variables, (2) assigning appropriate weights to each variable based on their biological relevance, and (3) performing a multicriteria evaluation to generate the ecophysiological suitability index. Raster layers representing environmental conditions were obtained from WorldClim v2.1 at a spatial resolution of 30 arcseconds (− 1 km^2^) (Fick and Hijmans, 2017). Specifically, we obtained the mean annual temperature (BIO1), maximum temperature of the warmest month (BIO5), and minimum temperature of the coldest month (BIO6), to capture the *Bd’s* thermal response curve (optima, critical maximum and critical minimum respectively). These layers were used to generate spatial representations of the selected ecophysiological variables (see Sect. "Review of Available Information About Bd Ecophysiology"). To derive continuous rasters for each physiological constraint, we applied conditional classification rules based on empirical data extracted from the literature (see Sect. "Review of Available Information About Bd Ecophysiology"). The conversion process was implemented in R v4.3.1 using the terra package.

To integrate the rasterized variables into a composite suitability index, we assigned weights based on their relative importance in *Bd* persistence and transmission. Given that *Bd’s* growth and survival are biologically relevant throughout the life cycle of the pathogen, growth and survival suitability received the highest weight 0.4. Zoospore density and zoospore activity duration were weighed at 0.2 each, as they significantly influence pathogen persistence and infection potential. The zoospore release amplitude and time to first zoospore release were lower 0.1, reflecting their role in modulating pathogen transmission but not necessarily persistence. This weighting scheme was informed by prior studies highlighting the influence of these variables on *the* epidemiology of *Bd* and considering its relevance to different life cycle stages of the fungus (Woodhams et al., 2008; Voyles et al., 2017; see Fig. 1).

The final ecophysiological suitability index was computed using a weighted linear combination approach in R, integrating the standardized raster layers as follows:

$$\text{Suitability} \text{index}=(\text{G}\times 0.4) + (\text{D}\times 0.2) + (\text{A}\times 0.2) + (\text{L}\times 0.1) - (\text{F}\times 0.1)$$

where.

G = Growth and survival suitability.

D = Zoospore density.

A = Zoospore activity duration.

L = Zoospore release amplitude.

F = Time to first zoospore release.

The resulting ecophysiological suitability index was normalized to a 0–1 scale using min–max normalization to ensure comparability between the present time and future climate change scenarios.

The model was validated using both the Boyce index (presence–presence validation) and receiver operating characteristic (ROC; presence–pseudoabsence validation) analyses, incorporating independent occurrence data for *Bd* in Mexico compiled from peer-reviewed literature. To ensure comprehensive coverage, occurrences were retrieved through keyword searches (e.g., *Batrachochytrium dendrobatidis*, chytridiomycosis) combined with geographic terms (e.g., México, State of Mexico, Yucatán) across Google Scholar, Scopus, and ResearchGate.

The Boyce index assesses the model’s ability to predict *Bd* presence by comparing observed occurrence frequencies to expected frequencies across habitat suitability bins. The predicted-to-expected ratio (F.ratio) was computed as follows:

$$F.\text{ratio}= \frac{F\text{obs}}{F\text{exp}}$$

where.

Fobs = the observed frequency of occurrences per bin.

Fexp = the proportional area of that bin in the study region.

The Boyce index (r) was calculated as the Spearman correlation between habitat suitability values and the frequency of observed presences across suitability classes (F.ratio), using the ecospat.boyce() function from the ecospat R package. Values range from − 1 to 1, where positive values indicate that presences occur more frequently in areas of high suitability than expected by chance (i.e., predictions better than random), values near zero indicate no predictive power, and negative values suggest that the model assigns higher suitability to areas where the species is absent (i.e., inverse predictions; Di Cola et al., 2017). ROC analysis was used to evaluate the ability of the model to discriminate between presence and pseudoabsence points, quantified by the area under the curve (AUC). AUC values above 0.7 indicate a fair predictive accuracy (Çorbacıoğlu and Aksel, 2023). The study area was partitioned into four geographically distinct subsets using Ward’s hierarchical clustering on latitude and longitude (squared Euclidean distance metric), implemented in Statgraphics v.19.6.04. The clusters obtained represent the Trans-Mexican Volcanic Belt (TMVB), Baja California Peninsula (BC), Chiapas, Tabasco, and Southern Veracruz subset (CTV) and the Mexican Altiplano and Sierra Madre Oriental subset (MA-SMO). These clusters were further aggregated into two broader subsets, Central-Southern Mexico and Northern Mexico, resulting in seven validation datasets (full dataset, four regional clusters, and two aggregated regions). For each subset, pseudoabsences were generated within the respective geographic range, ensuring that they occurred only in areas with *Bd* suitability < 0.2. Additionally, occurrence points were randomly assigned to four groups of equal sample sizes to evaluate predictive performance independent of spatial structure. Pseudoabsences were generated as in spatial partitioning and constrained to ecophysiological suitability values < 0.2. For all partitions, the true positive rate (TPR) and false positive rate (FPR) were calculated as follows:

$$TPR= \frac{TP}{TP+FN}$$

$$FPR= \frac{FP}{FP+TN}$$

where.

TP = true positives.

FP = false positives.

FN = false negatives.

TN = true negatives.

ROC curves and AUC values were computed using the pROC R package (roc() and auc() functions). This dual validation approach, which integrates spatial and random partitioning, ensured rigorous assessment of model performance across varying geographic and stochastic conditions, enhancing confidence in its predictive capabilities.

Then, we assess the impact of climate change on the potential distribution of *Bd*, replicating the modeling procedure using climatic projections for the years 2050 and 2070 under two shared socioeconomic pathways (SSP370 and SSP585). We selected SSP370 (Regional Rivalry) and SSP585 (Fossil-Fueled Development) to capture a plausible range of future climates, as representatives of intermediate- and high-end emission trajectories, respectively. SSP370 assumes slow economic growth, fragmented international cooperation, and limited climate mitigation, leading to moderate warming, whereas SSP585 envisions rapid fossil-fuel-driven development with minimal mitigation and extreme warming (Riahi et al., 2017; Lee et al., 2021). By contrasting these two narratives, our projections bracket both a mid-century “business-as-usual” pathway and a worst-case scenario, allowing us to assess the robustness of *Bd*’s potential range shifts under divergent socio-environmental futures. Future climate scenarios were obtained from WorldClim v2.1 based on simulations from the ACCESS-CM2 General Circulation Model, a widely used Earth system model that accounts for atmospheric, oceanic, and land-surface interactions (Bi et al., 2020).

### Risk for *Bd* Suitability in Mexico

To evaluate the potential risk of *Bd* for amphibians in Mexico, we conducted a spatial analysis incorporating NPAs and endemic amphibian distributions. Polygon layers representing federal and state-level NPAs were obtained from the Comisión Nacional para el Conocimiento y Uso de la Biodiversidad (CONABIO) geoportal. Additionally, we extracted distribution range polygons for 273 amphibian species endemics to Mexico from the International Union for Conservation of Nature (IUCN) Red List database. For each species, we compiled information on conservation status (as classified by the IUCN Red List categories), population trends (unknown, stable, or decreasing), family, and primary habitat type (freshwater, terrestrial, or terrestrial/freshwater). To quantify *Bd* suitability within these spatial units, we used the zonal statistics tool in QGIS to extract the mean, minimum, maximum, and standard deviation of *Bd* suitability values within each NPA polygon and each species distribution polygon. The suitability indices were subsequently analyzed to determine which species, and protected areas exhibited the highest vulnerability to *Bd*.

Furthermore, we examined patterns of *Bd* suitability across different families, conservation categories, habitat types, and population trends. Using the dynamic graphics function in Microsoft Excel, we computed mean *Bd* suitability scores across each categorical variable to identify groups at elevated risk. This allowed us to determine which families, conservation statuses, population trends and habitat types are most strongly associated with high *Bd* suitability, highlighting potential conservation priorities.

## Results

### Model Validation

#### Occurrence Points Used in Validation

**Figure 2:**
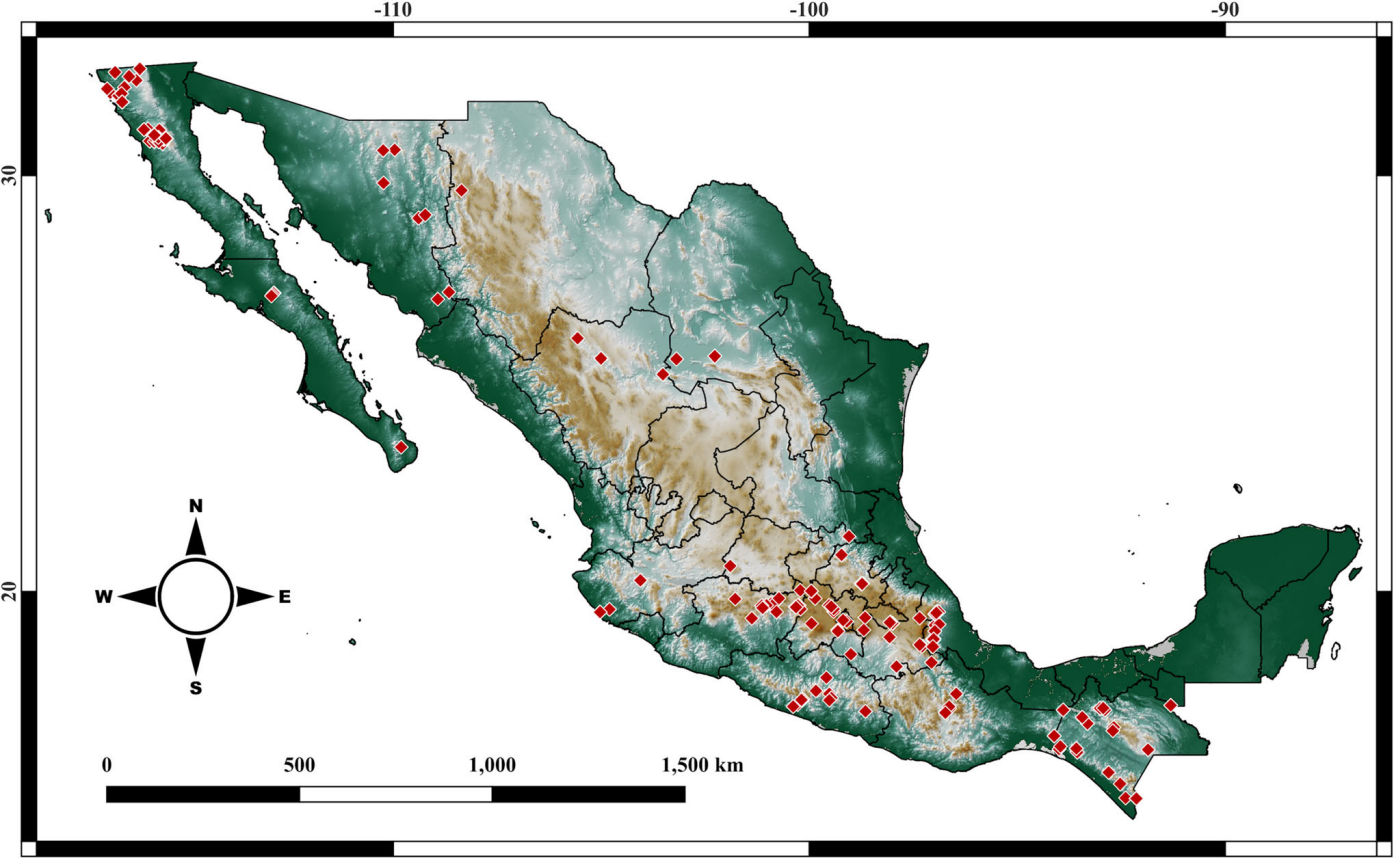
Occurrence points used in the validation were obtained from bibliographic sources (see Table S1). Note that twelve states in Mexico do not present *Bd* occurrence points and that most of the points are concentrated in central and southern Mexico.

The occurrence records used for the ecophysiological model validation revealed noteworthy patterns, as shown in Table S1. This review revealed its presence in 20 states and its impact on 99 species (Fig. 2). The states with the highest number of affected species were Chiapas (23 species), Estado de México (13 species), Veracruz (13 species), and Guerrero (15 species; see Table S1), while Baja California Sur, Guanajuato, Michoacán, Morelos, Tabasco, Tlaxcala, and Sonora had the lowest numbers. States without current *Bd* records include Aguascalientes, Campeche, Colima, Nayarit, Nuevo León, Querétaro, Quintana Roo, Sinaloa, Tamaulipas, and Yucatán (Fig. 2). *Batrachochytrium dendrobatidis* affects five microendemic species confined to highly specific habitats: *Ambystoma andersoni*, *Ambystoma mexicanum*, *Chiropterotriton chico*, *Chiropterotriton dimidiatus*, and *Dendrotriton megarhinhus*.

#### Presence–Presence Validation

The correlation coefficient of the Boyce index (r = 0.74) indicated a strong positive relationship between the observed species occurrence and predicted habitat suitability, confirming the ability of the model to distinguish suitable from unsuitable habitats. Habitat suitability (HS) ranged from 0.05 to 0.94, encompassing the full gradient of predicted environmental suitability. The F.ratio, which represents the ratio of observed to expected occurrences, varied across suitability intervals, providing detailed insights into model validation. At low suitability intervals (HS < 0.2), the F.ratio values were predominantly near or less than 1 (e.g., 0.000, 0.206), indicating that the model underestimated occurrences in marginal habitats, consistent with ecological expectations. For intermediate suitability intervals (0.3 ≤ HS ≤ 0.6), the F.ratio values stabilized at approximately 1 (e.g., 0.741, 1.032), demonstrating accurate predictions in moderately suitable habitats. Finally, in highly suitable habitats (HS > 0.6), the F.ratio values exceeded 1 (e.g., 1.794, 2.328), suggesting a slight overestimation of *Bd* presence in these regions.

#### Presence–Pseudoabsence Validation

When validation through AUC values was conducted using all occurrence points combined, the AUC was 0.7, indicating moderate predictive accuracy. Partitioning the data geographically revealed variable predictive performance across regions. The TMVB and BC subsets achieved the highest AUC values (0.8), demonstrating strong model accuracy in these regions. In contrast, the CTV exhibited the lowest AUC (0.6), indicating reduced predictive reliability. MA-SMO, the aggregated Central-Southern Mexico subset and the aggregated Northern Mexico subset achieved intermediate AUC values of 0.7. Regarding random partitioning, one subset presented an AUC value of 0.8, two had an AUC of 0.7, and the last one presented a reduced predictive reliability of 0.6.

### Ecophysiological Suitability for *Bd* in Mexico

#### Ecophysiological Variables

**Figure 3:**
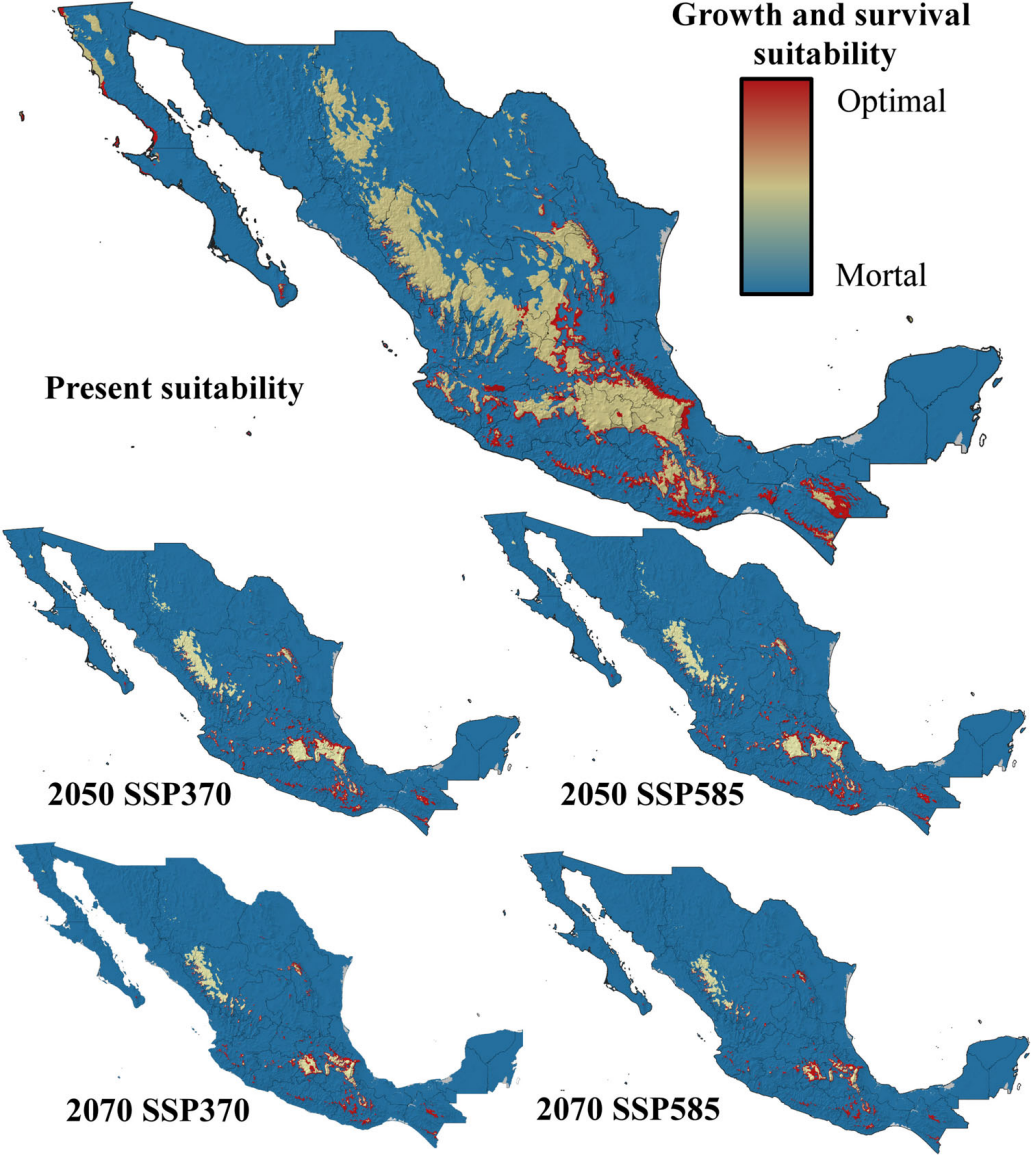
Growth and survival suitability for *Batrachochytrium dendrobatidis* in Mexico, at present time, for 2050 and 2070 under two different climate change scenarios, SSP370 and SSP585.

**Figure 4:**
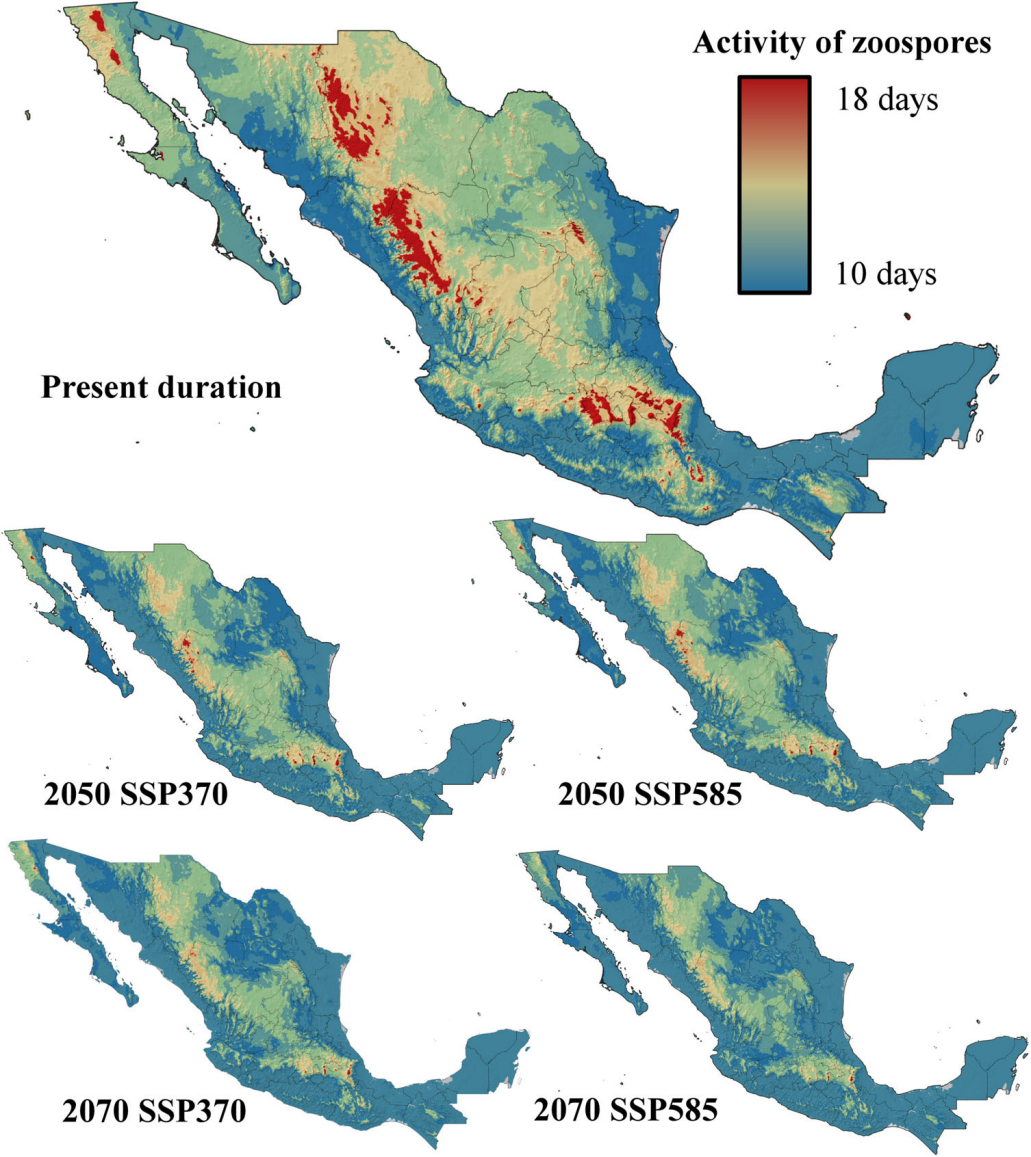
Activity duration of zoospores for *Batrachochytrium dendrobatidis* in Mexico, at present time, for 2050 and 2070 under two different climate change scenarios, SSP370 and SSP585.

**Figure 5:**
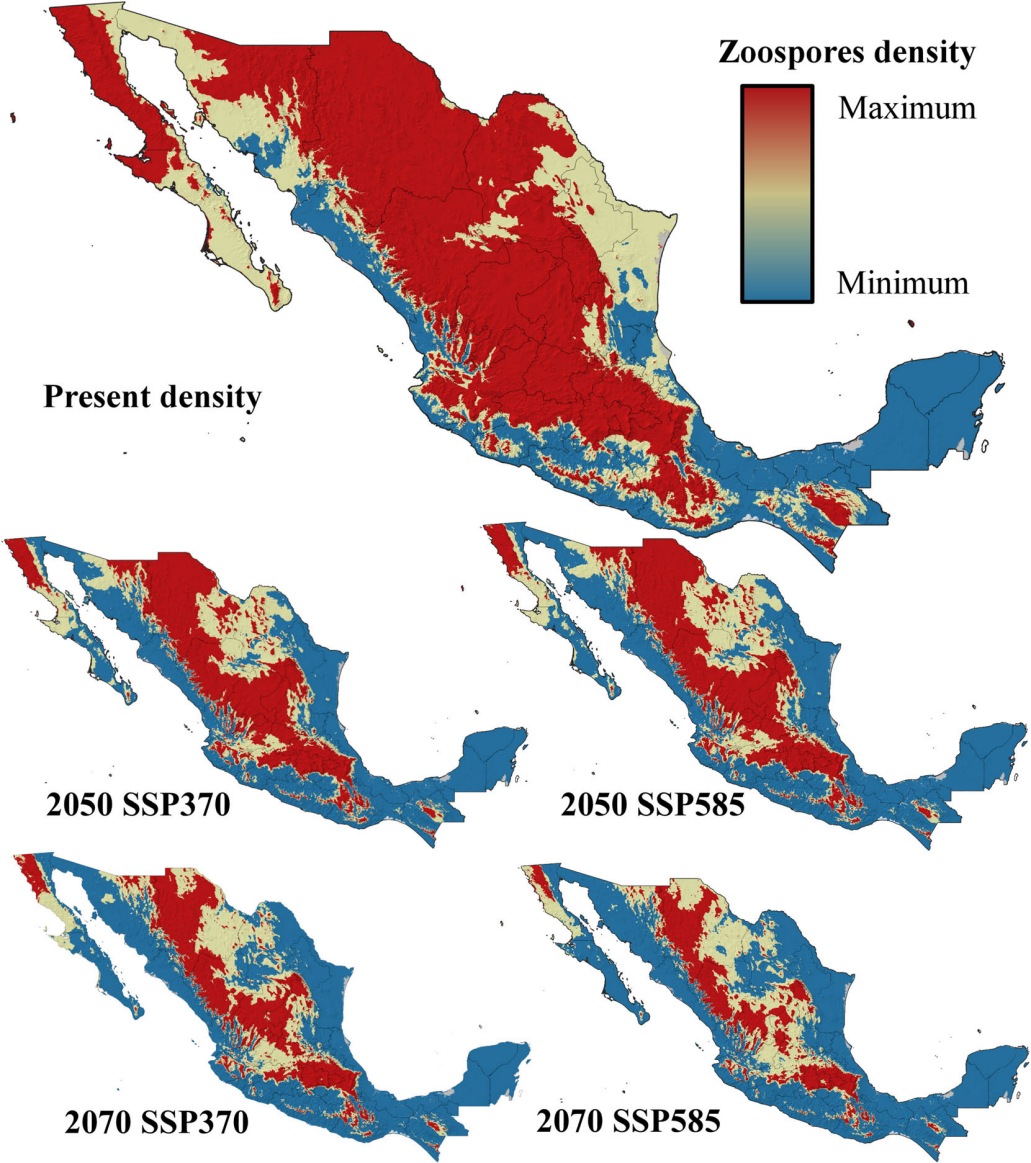
Zoospores density for *Batrachochytrium dendrobatidis* in Mexico, at present time, for 2050 and 2070 under two different climate change scenarios, SSP370 and SSP585.

**Figure 6:**
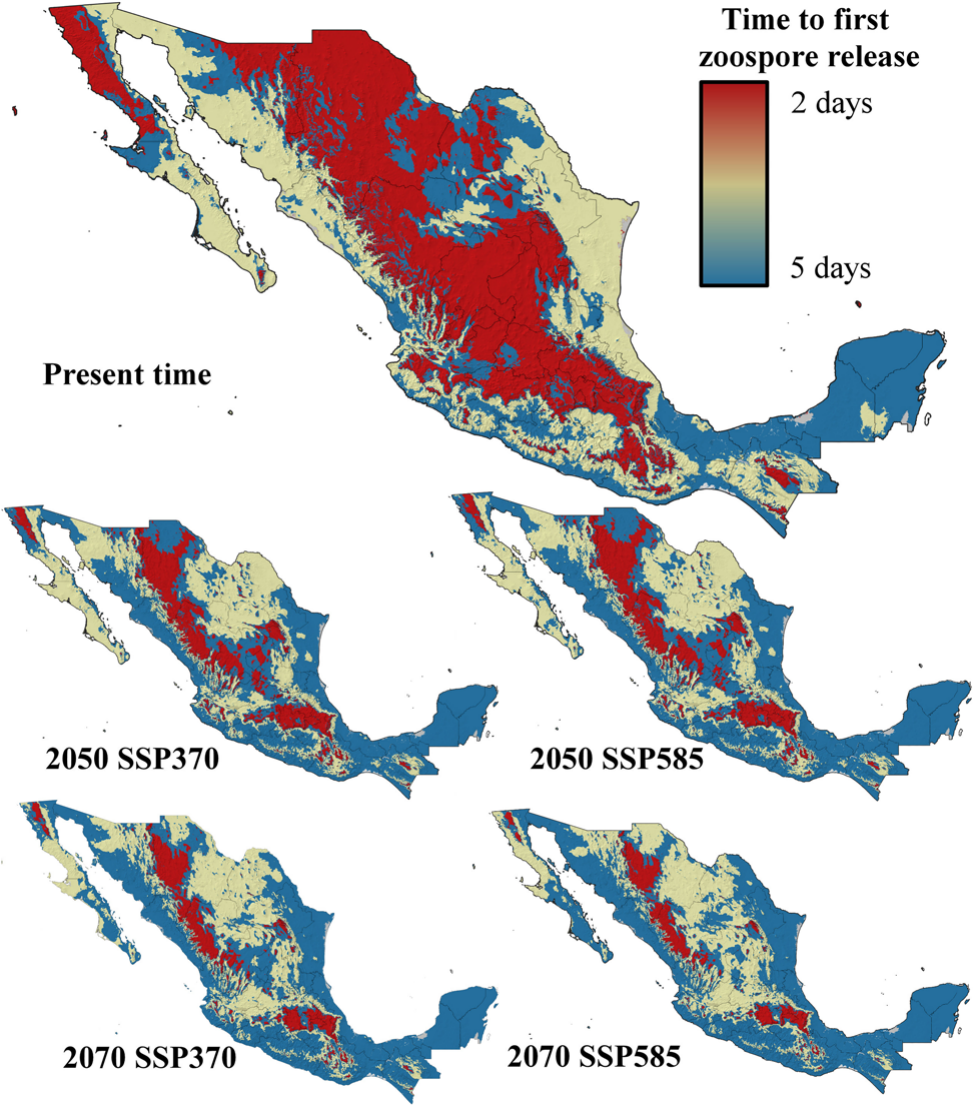
Time to first zoospore release for *Batrachochytrium dendrobatidis* in Mexico, at present time, for 2050 and 2070 under two different climate change scenarios, SSP370 and SSP585.

**Figure 7:**
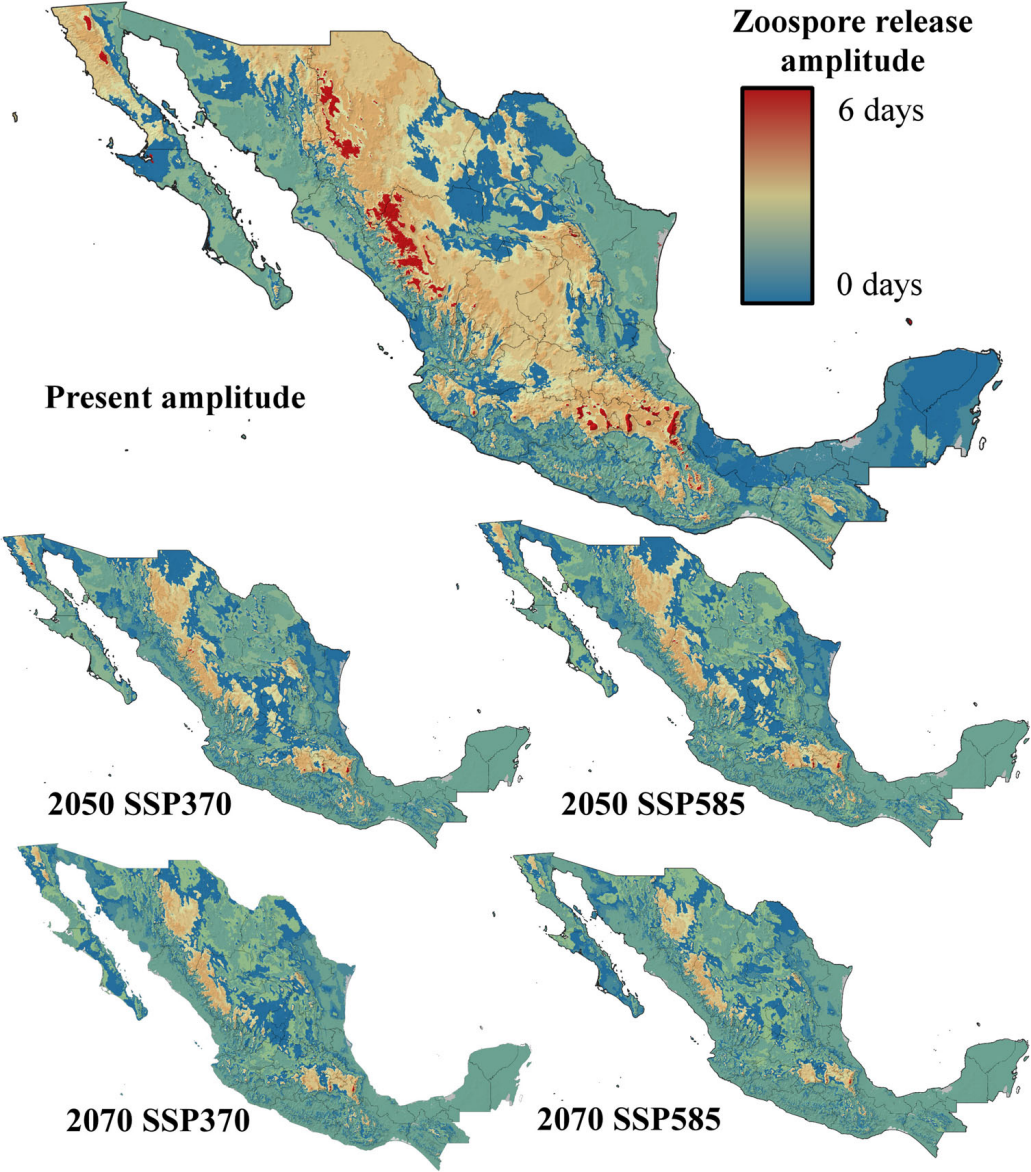
Zoospore release amplitude for *Batrachochytrium dendrobatidis* in Mexico, at present time, for 2050 and 2070 under two different climate change scenarios, SSP370 and SSP585.

Five ecophysiological variables were analyzed for current and future conditions under two contrasting climate change scenarios. The suitability for *Bd* growth and survival was highest in areas surrounding mountain ranges, with moderate suitability at higher elevations and low probabilities of success in lowlands and most coastal zones (Fig. 3). By 2050 and 2070, under both the SSP370 and SSP585 scenarios, the suitability for *Bd* growth and survival is projected to decrease significantly, with optimal conditions restricted to the foothills of Mexico’s highest mountain ranges (Fig. 3). Zoospore activity currently lasts up to 18 days in the highest mountain ranges in Mexico but no more than 10 days in most coastal zones and lowlands in northern and central Mexico, as well as at the highest elevations of the Yucatan Peninsula (Fig. 4). By 2050 and 2070, zoospore activity will persist for 18 days only in specific high-altitude areas, such as the TMVB in the State of Mexico, Mexico City, Tlaxcala, Puebla, and Veracruz; the SMO in western Durango; and the Sierra de San Pedro Mártir in Baja California. In the rest of the country, zoospore activity will be limited to less than two weeks (Fig. 4). Zoospore density currently reaches its maximum level across most of Mexico, except in coastal zones and the Yucatan Peninsula, where temperatures limit high densities (Fig. 5). However, the increase in temperature projected for 2050 and 2070 will constrain the maximum zoospore density to major mountain ranges under both climate change scenarios (Fig. 5). The time to the first zoospore release exhibited a complex pattern. While optimal conditions (as short as two days before the first release) are observed at higher elevations and in the MA, coastal zones also show varied results. Most coastal areas support rapid zoospore release, except for the Yucatan Peninsula, where *Bd* requires up to five days to initiate reproduction (Fig. 6). Under future climate change scenarios, the time to the first release will increase in coastal zones and MA (Fig. 6). Zoospore release amplitude is currently the longest (six days) in the TMVB, SMO, high-elevation areas of Baja California (e.g., Sierra Juárez and Sierra de San Pedro Mártir), and the eastern mountains of Oaxaca (Fig. 7). By 2050, climate change is projected to reduce this amplitude, with only the highest mountains in Mexico maintaining a six-day duration. Conversely, in regions such as the Yucatan Peninsula and Tabasco, where zoospore release is practically absent, climate change could facilitate a two-day release amplitude (Fig. 7). This pattern is expected to persist through 2070 (Fig. 7).

#### Present and Future Suitability for *Bd* in Mexico

**Figure 8:**
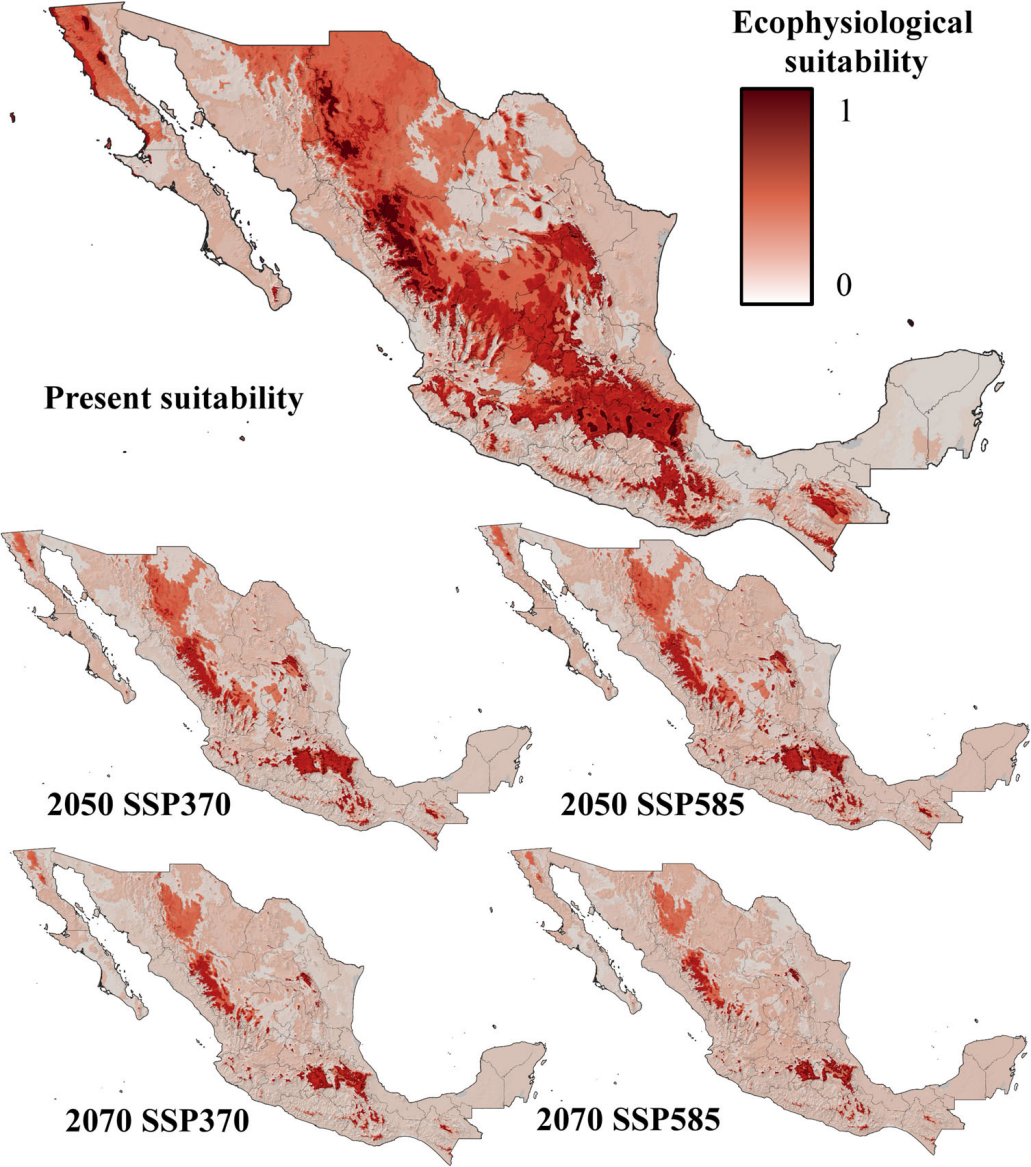
The ecophysiological suitability indices for *Batrachochytrium dendrobatidis* in Mexico for 2050 and 2070 under two different climate change scenarios, SSP370 and SSP585. Note the interesting reduction in *Bd’s* suitability over time, which was confined to mountain ranges over time.

The present distribution of *Bd* closely aligns with the behavior of individual ecophysiological variables, as mountain ranges in Mexico exhibit the highest suitability, whereas suitability in coastal zones and lowlands is null or close to zero (Fig. 8). Some states without documented *Bd* occurrence in the literature (see Sect. "Occurrence Points used in Validation"), including Zacatecas, San Luis Potosí, Tamaulipas, Coahuila, and the highest elevations of the Yucatan Peninsula, show high suitability (Fig. 8). By 2050, the suitability for B*d* is projected to decline across most of the country, with maximum suitability restricted to the highest elevations of Mexican mountain ranges (Fig. 8). This pattern was consistent under both the SSP370 and SSP585 scenarios (Fig. 8). By 2070, under both climate change scenarios, the highest suitability for *Bd* will be further confined to specific high-altitude areas in the Trans-Mexican Volcanic Belt (TMVB). The main differences between the times and climate change scenarios are presented in Fig. S1.

### Implications for Mexican Amphibian Conservation

#### Natural Protected Areas in Mexico at Risk for *Bd*

There are 203 federally protected NPAs in Mexico, encompassing 22,149,714.02 terrestrial hectares. Of these, 71 NPAs (35.0%) presented a habitat suitability index greater than 0.5 for *Bd*, covering a total area of 2,946,845.64 hectares, equivalent to 13.3% of the total federally protected area. Six federal NPAs exhibited a complete suitability score (value of 1), indicating maximum risk for *Bd* occurrence. These include Nevado de Toluca (State of Mexico), Iztaccíhuatl-Popocatépetl (State of Mexico, Morelos and Puebla), Cofre de Perote (Veracruz), Cumbres del Ajusco (Mexico City), Pico de Orizaba (Veracruz and Puebla), and Arrecife Alacranes (Yucatán). In contrast, 10 federal NPAs showed null suitability for *Bd*, including areas such as Bala’an K’aax, Chan-Kin, and Tulum in the Yucatan Peninsula, as well as various islands and coastal regions. For detailed information on the mean habitat suitability, minimum and maximum suitability values, and standard deviations for each federal NPA, see Table S2.

At the state level, Mexico has 299 NPAs, protecting a total of 3,282,299.51 hectares. Of these, 158 NPAs (52.8%) exhibited an ecophysiological suitability index greater than 0.5 for *Bd*, representing 1,114,672.05 hectares or 33.96% of the total area protected by state NPAs. Five state NPAs show absolute suitability for *Bd*: Centro Ceremonial Mazahua and Santuario del Agua Presa Brockman y Victoria in the State of Mexico, Cerro El Potosí (Nuevo León), Quebrada de Santa Bárbara (Michoacán), and San Pedro en el Monte (Querétaro). Conversely, 14 state NPAs presented null suitability for *Bd*, including areas such as Kabah (Quintana Roo), Arroyo Moreno (Veracruz), and Estero El Salado (Jalisco). The detailed statistics, including the distributions of habitat suitability values for state NPAs, can be found in Table S3.

#### Endemic Amphibians in Mexico at Risk for *Bd*

Among the 273 amphibian species endemic to Mexico, 158 species exhibited a mean habitat suitability for *Bd* above 0.5 across their geographic distributions, representing 57.87% of the endemic amphibian species. For *Bd*, 12 species, three ambystomatids (*Ambystoma leorae*, *A. rivulare*, and *A. altamirani*) and nine plethodontids, have an ecophysiological suitability of 0.9 for *Bd* in their habitats. All these species are categorized under some level of risk according to the IUCN Red List and exhibit declining populations (Table S4). No endemic amphibian species show null suitability for *Bd* within their geographic distribution; however, five species have values lower than 0.1: *Incilius pisinnus*, *Eleutherodactylus colimotl*, *Bolitoglossa veracrucis*, *Craugastor silvicola*, and *Craugastor yucatanensis*.

**Figure 9:**
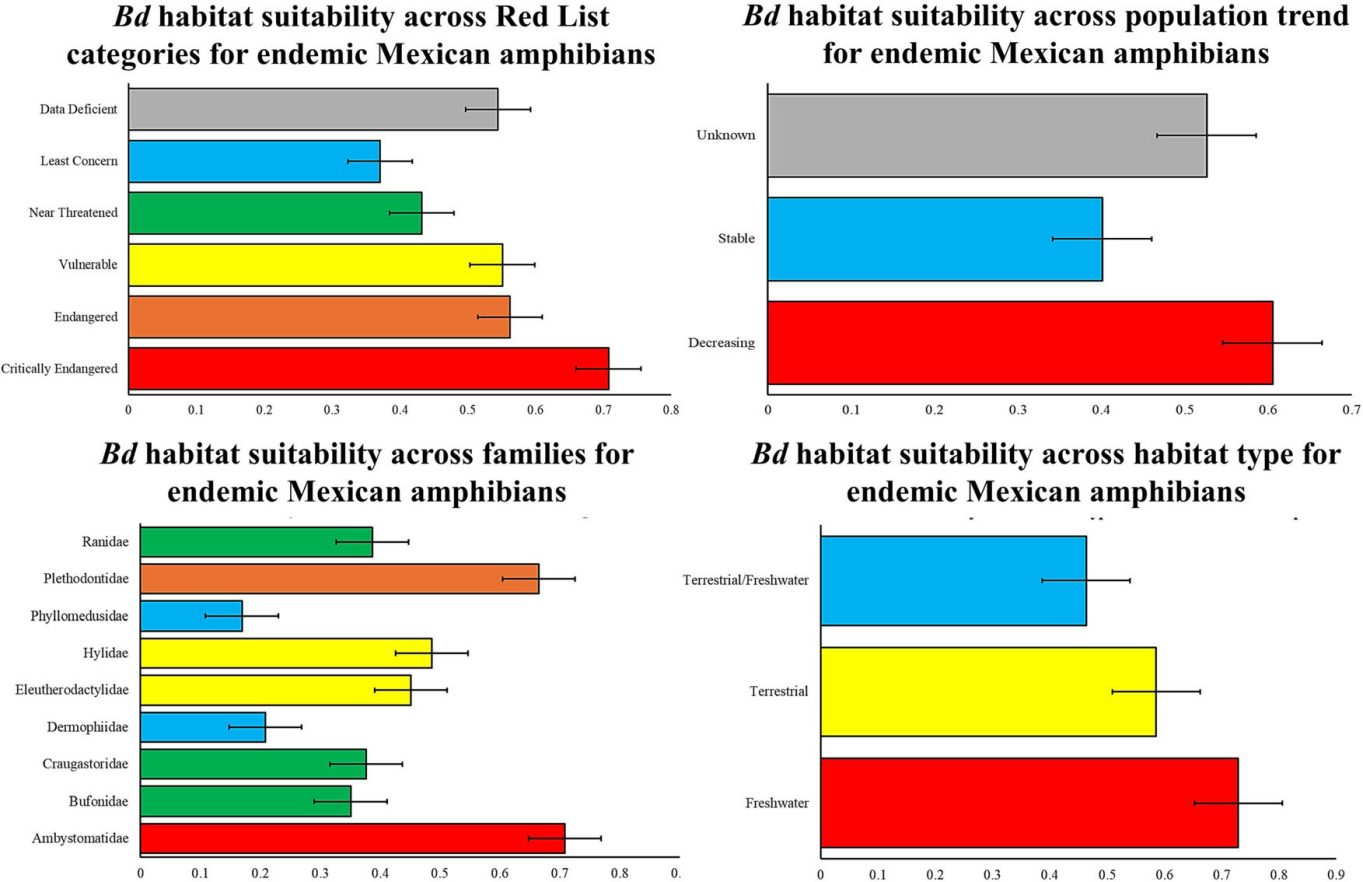
Ecophysiological suitability of *Batrachochytrium dendrobatidis* for the distribution of endemic Mexican amphibians classified according to four criteria: International Union for Conservation of Nature (IUCN) Red List categories, population trends, taxonomic families, and habitat types. The bars represent the mean suitability values ± standard errors.

In terms of IUCN risk categories, species classified as "critically endangered" had the highest average suitability for *Bd* (0.7), followed by those categorized as "endangered" (0.6) or "vulnerable" (0.6). In contrast, species listed as "Least Concern" and "Data Deficient" exhibited lower average suitability values. Regarding population trends, species with declining populations had higher suitability indices (0.6) than did those with stable populations or unknown trends. At the family level, the highest average suitability values were observed for Plethodontidae (0.7) and Ambystomatidae (0.7), while families such as Dermophiidae and Phyllomedusidae had the lowest values (Fig. 9). Finally, classification by habitat type indicated that species associated with aquatic habitats had the highest average suitability (0.72), followed by those inhabiting terrestrial environments (0.58), whereas species inhabiting mixed environments had the lowest values (0.46; Fig. 9).

## Discussion

### Model Validation

The validation methods used confirmed the model’s ability to distinguish suitable from unsuitable habitats, particularly in highly suitable areas (HS > 0.6), where predictions align well with observed occurrences. However, the model showed limitations in terms of marginal habitats (HS < 0.2), where occurrences were underestimated, and in regions such as CTV, where AUC values (0.6) indicate reduced predictive reliability. The use of a mechanistic model demonstrated good resolution for explaining *Bd* ecophysiological suitability in Mexico, enabling more accurate predictions of pathogen dynamics under both current and future conditions and avoiding the existing limitations of correlative models. Compared to Bolom-Huet et al. (2019), who documented *Bd* in 14 states in Mexico and 78 species, our updated review of occurrence data for validation reveals that *Bd* now inhabits 20 states and affects 99 species. This expansion reveals the growing intensity of research efforts across Mexico.

### Ecophysiological Suitability Index Present, Future and Under Different Climate Change Scenarios

The ecophysiological suitability index revealed high suitability for *Bd* across most of Mexico, with only coastal zones and lower-altitude areas being less favorable for fungal occurrence and propagation under current conditions. Mountain ranges are the most favorable environments for *Bd* in Mexico, and this pattern is expected to persist into the future, even under contrasting climate change scenarios (Fig. 8). In Mexico, mountain ranges are especially valuable for amphibian conservation since these regions harbor high taxonomic diversity (Ramírez-Bautista et al., 2023), which implies an urgent need for conservation strategies against *Bd*. Interestingly, the index also highlights the high suitability of *Bd* for Mexican states where the pathogen has not yet been recorded, including Zacatecas, San Luis Potosí, Tamaulipas, and Coahuila, emphasizing the need for continued monitoring and recording of *Bd* occurrences in understudied regions.

Under future climate scenarios (2050 and 2070), *Bd’s* suitability is projected to decrease in many areas outside mountain ranges. However, certain regions, such as the Pacific coast, coasts in southern Veracruz, and the Yucatan Peninsula, may experience an increase in suitability, potentially enabling the fungus to establish itself in areas currently less favorable. Conversely, not all coastal zones are projected to follow this trend, as most of the Gulf of Mexico coastlines are expected to experience a reduction in suitability for chytridiomycosis.

Regarding the climate change scenarios evaluated, differences between SSP370 and SSP585 are relatively minor in 2050, with most reductions in *Bd* suitability occurring in Baja California Sur, Sonora, and parts of the Mexican Altiplano—particularly in Zacatecas—while slight increases appear in the Sierra de Baja California (Fig. S1). However, by 2070, the divergence between scenarios becomes more pronounced. In the southern Altiplano, *Bd* suitability increases under SSP585 scenarios whereas in the north, that scenario predicts reductions in the eastern sector and increases in the west. Notably, under SSP585, *Bd* suitability becomes more restricted to high-elevation areas such as the Trans-Mexican Volcanic Belt and mountain systems in Baja California (Fig. S1). These patterns are driven primarily by rising temperatures. *Batrachochytrium dendrobatidis* exhibits reduced viability at temperatures above its thermal optimum, and its survival sharply declines under prolonged exposure to temperatures exceeding 30 °C (Piotrowski et al., 2004). According to WorldClim v2.1 projections, temperature increases are expected throughout the twenty-first century, with the magnitude of change varying by scenario. While low-end scenarios like SSP1-2.6 project a 0.21–1.0 °C increase, intermediate pathways such as SSP370 forecast warming of 2.0–3.7 °C, and high-end scenarios like SSP585 project increases of 2.4–4.8 °C (Riahi et al., 2017; Lee et al., 2021). These differences explain the progressive contraction of suitable habitat for *Bd* under future scenarios, with more severe reductions expected under SSP585. Thus, our results suggest that global warming could act as a limiting factor for *Bd* proliferation in Mexico, although high elevation refugia may remain vulnerable under all scenarios. Our ecophysiological suitability index aligns with previous distribution models for *Bd* in Mexico, such as the model by Bolom-Huet et al. (2019), which employed the MAXENT algorithm using bioclimatic variables, evapotranspiration, and the human footprint; and, to a lesser extent, the model by Jacinto-Maldonado et al. (2023), which relied on water pollution. The latter utilized an algorithm based on the relationship between *Bd* presence and an innovative variable that integrates amphibian family distribution and water quality metrics. All three models underscore the vulnerability of mountain ranges in Mexico to *Bd*, once again highlighting the importance of these regions in developing strategies to mitigate the prevalence of the pathogen. However, notable discrepancies emerge between the models. While both our index and the model by Bolom-Huet et al. (2019) suggest that most coastal zones are currently unsuitable for *Bd*, Jacinto-Maldonado et al. (2023) indicate high suitability for the fungus along the Pacific and Gulf of Mexico coasts. These differences highlight the multifactorial nature of *Bd* incidence. Although environmental variables seem to better reflect the ecophysiological suitability of the fungus, incorporating insights into amphibian diversity, water quality, and their interaction with *Bd* prevalence provides valuable perspectives. This integrative approach may help elucidate potential mechanisms for *Bd* dispersion, even in areas where environmental and physiological constraints might otherwise limit its prevalence.

### Implications in Conservation

The projected reduction in *Bd’s* ecophysiological suitability in some regions provides a valuable framework for prioritizing conservation efforts. By focusing on areas where suitability is expected to persist or increase by 2050 and 2070, such as the TMVB, SMO, and Sierra Madre del Sur, resources can be directed to mitigate the greatest future impacts. This approach addresses one of the most pressing challenges in biological conservation: the allocation of limited resources across regions (Wilson et al., 2006). Given the finite resources available, it is critical to balance immediate interventions in currently impacted areas with proactive measures taken in regions predicted to face increased risk. Our results suggest prioritizing conservation efforts in regions with a high presence of NPAs to take advantage of the fact that there is already a territorial order, particularly targeting Ambystomatids and Plethodontidae species categorized as “Critically Endangered,” “Endangered,” or “Vulnerable” by the IUCN Red List. Effective management of freshwater bodies, including pollutant mitigation, should be a priority in these regions. Additionally, monitoring efforts must urgently expand to Mexican states lacking *Bd* records but showing high ecophysiological suitability, as identified in our model. Implementing an independent prioritization strategy, as recommended by Cattarino et al. (2015), offers a cost-effective pathway for amphibian conservation in Mexico. This approach enables conservationists to target high-risk areas first while maintaining flexibility in addressing emerging threats.

Understanding *Bd*’s life cycle is essential for designing effective mitigation strategies, particularly those targeting vulnerable stages such as zoospores (Ward and Brown, 2004; Sentenac et al., 2024). This knowledge becomes especially relevant when linked to the ecological conditions favoring those stages, as revealed by our ecophysiological suitability model. The ecophysiological variables used to construct the suitability index (Figs. 4, 5, and 7) suggest that mountain ranges in Mexico are particularly suitable regions for implementing such strategies, as they exhibit maximum zoospore density, activity duration, and release amplitude, making them critical focal points for conservation efforts directed to attack zoospores. Conversely, ecological refuges, specifically hotspot shelters, as described by Waddle et al. (2024), may be more effective in regions where *Bd* growth and survival peak, such as the edges of mountain ranges and throughout most of the territory of Chiapas. Similarly, regions such as the Yucatan Peninsula, the southern coast of Veracruz, and the Pacific Coast, where the zoospore release amplitude is projected to increase by 2050, present another challenge. Here, hotspot shelters (see Garner et al., 2016) could be especially effective due to the relatively high heat tolerance of coastal amphibian species (Snyder and Weathers, 1975), which makes temperature-based treatments more feasible. By combining these ecological insights with tailored interventions, conservation efforts can strategically disrupt *Bd’s* life cycle, reduce its spread, and protect amphibian populations in regions at highest risk.

Building on our quantification of *Bd* suitability within Mexico’s protected areas (Tables S2–S3), we highlight several key conservation implications. First, the finding that over one-third of federal NPAs and more than half of state NPAs exceed a suitability threshold of 0.5 underscores the urgency of integrating chytrid surveillance into routine management plans. NPAs with maximum suitability (e.g., Nevado de Toluca, Iztaccíhuatl–Popocatépetl, Cofre de Perote) should be prioritized for systematic pathogen monitoring, biosecurity protocols, and habitat management to reduce transmission risk. Second, state NPAs with absolute suitability (e.g., Centro Ceremonial Mazahua, Cerro El Potosí) represent critical hotspots where targeted interventions—such as environmental DNA screening of water bodies and amphibian health assessments—could be piloted. Conversely, NPAs with null suitability may serve as refugia and inform landscape-level connectivity planning to bolster populations migrating from high-risk zones. Moreover, among the 273 endemic amphibian species analyzed (Table S4), 158 species (57.9%) exhibit mean suitability for *Bd* > 0.5, with critically endangered Ambystomatidae and Plethodontidae showing the highest risk scores. Species inhabiting aquatic habitats also face elevated suitability (mean = 0.72). These results highlight priority taxa for focused conservation actions, such as ecological refugees, and underscore the value of aligning species-level monitoring with protected-area management. Finally, integrating our ecophysiological suitability maps for *Bd* and species-level risk profiles within existing frameworks enables resource-efficient allocation of monitoring efforts and supports adaptive management strategies to safeguard Mexico’s endemic amphibians against *Bd* under both current and future climate scenarios.

### Limitations and Uncertainties

Mechanistic models, by design, depend on detailed, species‐specific data on physiological responses to environmental drivers (Dormann et al., 2012). In our case, *Bd*’s thermal biology is exceptionally well characterized, enabling robust parameterization of life‐history traits. However, many pathogens lack sufficient experimental data on temperature, moisture, or other physiological constraints, limiting the mechanistic approach’s applicability. Users should ensure that underlying trait data are derived from representative strains and ecological contexts to avoid biased projections.

Conversely, correlative models require only occurrence records and environmental layers but assume species–environment equilibrium and may conflate host, vector, and pathogen distributions (Václavík & Meentemeyer, 2009). While simpler to implement, these models risk mis‐attributing suitable habitat when key drivers—such as dispersal by human trade or water quality—are omitted. Our mechanistic index, based entirely on temperature, aligns closely with Bolom-Huet et al. (2019) but diverges from Jacinto-Maldonado et al. (2023), which integrated amphibian family distributions and pollution metrics. These differences highlight that factors beyond temperature (e.g., host community composition, hydrology, and anthropogenic disturbance) can promote or limit *Bd* establishment.

Finally, climate projections carry inherent uncertainties. We modeled two high‐emission pathways (SSP370, SSP585) using ACCESS‐CM2 outputs; other scenarios (e.g., SSP1-2.6) may yield less drastic warming and correspondingly different suitability patterns. Future refinements could incorporate hydrological models, land‐use change, and host‐pathogen interaction networks to provide more comprehensive forecasts. Despite these uncertainties, our ecophysiological framework offers a transparent, reproducible foundation for predicting pathogen suitability under environmental change.

## Conclusions

Our study highlights the utility of mechanistic models as tools for understanding the ecological dynamics of *Batrachochytrium dendrobatidis* and identifying priority regions for conservation efforts. The integration of ecophysiological data allowed us to pinpoint areas where different life cycle stages reached their most suitable conditions, offering targeted opportunities for intervention. Despite the inherent challenges in modeling pathogen distributions, our findings provide a solid framework for developing mitigation strategies, such as the establishment of ecological refuges and the use of biofilms to disrupt zoospore propagation. By addressing both current and future risks, this work underscores the need for proactive conservation measures that incorporate the ecophysiological complexity of *Bd* and its interactions with changing environmental conditions.

## Supplementary Information

Below is the link to the electronic supplementary material.

Supplementary file1 (PDF 3198 kb)
