# Supplementary material for: Ecophysiological Suitability of Batrachochytrium dendrobatidis in Mexico
Source: Ecohealth. 2025 Jul 15;22(4):533–52. doi: 10.1007/s10393-025-01734-w (PMC12628473; doi:10.1007/s10393-025-01734-w)
Supplement: Supplementary file 1 — Supplementary file1 (PDF 3198 kb) [file 10393_2025_1734_MOESM1_ESM.pdf]

Suplementary Material: Ecophysiological Suitability of *Batrachochytrium dendrobatidis* in Mexico

Table S1. Independent occurrence points for *Batrachochytrium dendrobatidis* in Mexico, used for validation of the ecophysiological suitability index.

Species	State	Source
Agalychnis moreletii	Guerrero, Chiapas	Frías-Alvarez et al,. 2008, Quintero Díaz et al., 2013
Ambystoma altamirani	Estado de México	Basanta et al., 2021a, Frías-Alvarez et al,. 2008
Ambystoma andersoni	Michoacán	Basanta et al., 2019
Ambystoma flavipiperatum	Jalisco	Basanta et al., 2019
Ambystoma granulosum	Estado de México, Michoacán	Frías-Alvarez et al,. 2008
Ambystoma mexicanum	Ciudad de México	García-Feria et al., 2019
Ambystoma ordinarium	Michoacán	Méndoza-Almeralla et al., 2023
Ambystoma rivulare	Estado de México, Michoacán	Basanta et al., 2019, Nava-González et al., 2021, Frías-Alvarez et al,. 2008
Ambystoma velasci	Puebla	Frías-Alvarez et al,. 2008
Anaxyrus boreas	Baja California	Peralta García et al., 2018
Anaxyrus californicus	Baja California	Peralta García et al., 2018
Anaxyrus cognatus	Durango	Hernández-Martínez et al., 2019
Anaxyrus debilis	Durango	Hernández-Martínez et al., 2019
Anaxyrus punctatus	Coahuila de Zaragoza	Hernández-Martínez et al., 2019
Aquiloeurycea cephalica	Estado de México, Hidalgo	Van Rooij et al., 2011
Bolitoglossa lincolni	Chiapas	Cabrera-Hernández, 2012
Bolitoglossa lincolni	Chiapas	Quintero Díaz et al., 2013
Bolitoglossa occidentalis	Chiapas	Basanta et al., 2021b
Bolitoglossa rufescens	Veracruz, Chiapas	Basanta et al., 2021b, Van Rooij et al., 2011, Quintero Díaz et al., 2013
Charadrahyla taeniopus	Veracruz	García-Feria et al., 2019
Chiropterotriton chico	Hidalgo	Basanta et al., 2021b
Chiropterotriton dimidiatus	Hidalgo	Cheng et al., 2011
Chiropterotriton multidentatus	Hidalgo	Cheng et al., 2011
Craugastor loki	Veracruz	Murrieta-Galindo et al., 2014
Craugastor pygmaeus	Guerrero	Familiar-López, 2007
Craugastor rhodopis	Veracruz	Murrieta-Galindo et al., 2014
Craugastor saltator	Guerrero	Lips et al., 2004
Dendropsophus ebraccatus	Chiapas	Quintero Díaz et al., 2013
Dendrotriton megarhinus	Chiapas	Quintero Díaz et al., 2013
Dendrotriton xolocalcae	Chiapas	Quintero Díaz et al., 2013
Duellmanohyla schmidtorum	Chiapas, Chiapas	Quintero Díaz et al., 2013
Ecnomiohyla miotympanum	Veracruz	Murrieta-Galindo et al., 2014

Eleutherodactylus cystignathoides	Veracruz	García-Feria et al., 2019
Eleutherodactylus dilatus	Guerrero	Basanta et al., 2021b
Eleutherodactylus nitidus	Guerrero	Familiar-López, 2007
Eleutherodactylus pipilans	Guerrero	Familiar-López, 2007
Exerodonta melanomma	Guerrero	Frías-Alvarez et al,. 2008
Exerodonta sumichrasti	Guerrero	Familiar-López, 2007
Gastrophryne mazatlanensis	Sonora	Jacinto-Maldonado et al., 2024
Gastrophryne olivacea	Durango	Hernández-Martínez et al., 2019
Hyalinobatrachium fleischmanni	Veracruz	García-Feria et al., 2019
Hyla arenicolor	Estado de México, Puebla	García-Feria et al., 2019
Hyla euphorbiacea	Oaxaca	Frías-Alvarez et al,. 2008
Hyla eximia	Estado de México, Guanajuato	Frías-Alvarez et al,. 2008, García-Feria et al., 2019, Leyte–Manrique et al., 2023
Hyla plicata	Estado de México, Michoacán	Nava-González et al., 2020
Hyla walkeri	Chiapas	Quintero Díaz et al., 2013
Hyla wrightorum	Chihuahua	Basanta et al., 2021b
Hypopachus barberi	Chiapas	Quintero Díaz et al., 2013
Incilius macrocristatus	Oaxaca	Cabrera-Hernández, 2012
Incilius macrocristatus	Chiapas	Quintero Díaz et al., 2013
Incilius occidentalis	Guerrero	Basanta et al., 2021b
Incilius tutelarius	Chiapas	Bolom-Huet et al., 2023
Incilius valliceps	Chiapas	Quintero Díaz et al., 2013, Cabrera-Hernández, 2012
Isthmura bellii	Estado de México, Michoacán, Guerrero	Nava-González et al., 2020, Basanta et al., 2021b
Leptodactylus fragilis	Tabasco	García-Feria et al., 2019
Leptodactylus melanonotus	Sonora, Jalisco	Basanta et al., 2021b, Cortes, 2014
Parvimolge townsendi	Veracruz	Cheng et al., 2011
Plectrohyla ixil	Chiapas	Quintero Díaz et al., 2013
Plectrohyla lacertosa	Chiapas	Quintero Díaz et al., 2013
Plectrohyla matudai	Chiapas, Oaxaca	Quintero Díaz et al., 2013, Cabrera-Hernández, 2012
Plectrohyla sagorum	Chiapas	Quintero Díaz et al., 2013
Pseudacris cadaverina	Baja California	Peralta García et al., 2018
Pseudacris regilla	Baja California, Baja California Sur	Peralta García et al., 2018, Luja et al., 2012
Pseudoeurycea firscheini	Puebla, Veracruz	Van Rooij et al., 2011, Basanta et al., 2021b
Pseudoeurycea juarezi	Oaxaca	Basanta et al., 2021b
Pseudoeurycea leprosa	Estado de México, Tlaxcala	Van Rooij et al., 2011, Méndoza-Almeralla et al., 2016
Pseudoeurycea longicauda	Michoacán	Nava-González et al., 2020
Pseudoeurycea nigromaculata	Veracruz	Basanta et al., 2021b, Cheng et al., 2011

Pseudoeurycea smithi	Oaxaca	Basanta et al., 2021b, Cheng et al., 2011
Ptychohyla erythromma	Guerrero	Lips et al., 2004
Ptychohyla euthysanota	Chiapas, Oaxaca	Quintero Díaz et al., 2013, Cabrera-Hernández, 2012
Ptychohyla leonhardschultzei	Guerrero	Familiar-López, 2007
Rana berlandieri	Durango, Veracruz, Chiapas, Oaxaca	Hernández-Martínez et al., 2019, García-Feria et al., 2019, Murrieta-Galindo et al., 2014, Quintero Díaz et al., 2013, Cabrera-Hernández, 2012
Rana catesbeiana	Baja California, Durango	Peralta García et al., 2018, Hernández-Martínez et al., 2019
Rana draytonii	Baja California	Peralta García et al., 2018
Rana forreri	Guerrero	Familiar-López, 2007
Rana maculata	Chiapas	Basanta et al., 2021b, Quintero Díaz et al., 2013
Rana megapoda	Estado de México	Frías-Alvarez et al,. 2008
Rana montezumae	Ciudad de México, Estado de México, Michoacán	Frías-Alvarez et al,. 2008, Basanta et al., 2021a, Nava-González et al., 2020
Rana neovolcanica	Estado de México, Michoacán	Frías-Alvarez et al,. 2008, Nava-González et al., 2020
Rana sierramadrensis	Guerrero	Familiar-López, 2007, Familiar-López, 2007
Rana spectabilis	Estado de México, Michoacán, Morelos, Puebla	Nava-González et al., 2020, Nava-González et al., 2021, Frías-Alvarez et al,. 2008, García-Feria et al., 2019
Rana tarahumarae	Sonora	Basanta et al., 2021b
Rana yavapaiensis	Sonora	Jacinto-Maldonado et al., 2024
Rheohyla miotympanum	Veracruz	García-Feria et al., 2019
Sarcohyla arborescandens	Puebla	Luría-Manzano et al., 2011
Sarcohyla bistincta	Michoacán	Nava-González et al., 2020
Sarcohyla pentheter	Guerrero	Familiar-López, 2007
Scaphiopus couchii	Sonora	Jacinto-Maldonado et al., 2024
Smilisca baudinii	Tabasco	García-Feria et al., 2019
Smilisca baudinii	Chiapas	Quintero Díaz et al., 2013, Cabrera-Hernández, 2012
Smilisca fodiens	Sonora, Jalisco	Basanta et al., 2021b, Basanta et al., 2021b, Cortés, 2014
Thorius pennatulus	Veracruz	Cheng et al., 2011
Tlalocohyla loquax	Chiapas	Quintero Díaz et al., 2013
Tlalocohyla smithii	Jalisco	Jacinto-Maldonado et al., 2020

References Table S1

Basanta MD, Betancourt-León O, Chávez OL, Pérez-Torres A, Rebollar EA, Martínez-Ugalde E, Ávila Akerberg VD, González-Martínez TM, Vázquez-Trejo M, Parra-Olea G (2021a) *Batrachochytrium dendrobatidis* occurrence in dead amphibians of central mexico: a report of *Ambystoma altamirani* and *Lithobates montezumae*. Revista Latinoamericana de Herpetología 4:173–177. DOI: 10.22201/fc.25942158e.2021.1.209 [Online May 25, 2011]

Basanta MD, Byrne AQ, Rosenblum EB, Piovia-Scott J, Parra-Olea G (2021b) Early presence of *Batrachochytrium dendrobatidis* in Mexico with a contemporary dominance of the global panzootic lineage. Molecular Ecology 30: 424–437. DOI: 10.1111/mec.15733 [Online December 17, 2020]

Basanta MD, Calzada-Arciniega RA, Jímenez-Velázquez G, Arias-Balderas SF, Ibarra-Reyes AA, Medina-Rangel G, Suazo-Ortuño I, Ochoa-Ochoa LM, Parra-Olea G (2019) Detection of *Batrachochytrium dendrobatidis* in Threatened Endemic Mole Salamanders (Ambystoma) in Mexico. Herpetological Review 50:493–495.

Bolom-Huet R, Pineda E, Andrade-Torres A, Díaz-Fleischer F, Muñoz AL, Galindo-González J (2023) Chytrid prevalence and infection intensity in treefrogs from three environments with different degrees of conservation in Mexico. Biotropica 55:318–328. DOI: 10.1111/btp.13186 [Online December 12, 2022]

Cabrera-Hernández R (2012) Evaluación de la presencia del hongo *Batrachochytrium dendrobatidis*, en poblaciones de anfibios en área cero extinción (aze) en Oaxaca y Chiapas, México. Lacandonia, 6:7-16.

Cheng TL, Rovito SM, Wake DB, Vredenburg VT (2011) Coincident mass extirpation of neotropical amphibians with the emergence of the infectious fungal pathogen *Batrachochytrium dendrobatidis*. Proceedings of the National Academy of Sciences of the United States of America, 108:9502–9507. DOI: 10.1073/pnas.1105538108 [Online June 07, 2011]

Cortés J (2014) Presencia del hongo quitridio *Batrachochytrium dendrobatidis* en zonas conservadas y fragmentadas en comunidades de anfibios de la Cuenca Hidrológica de Cuixmala, en el Estado de Jalisco, México. Thesis, Facultad de Medicina Veterinaria y Zootecnia, Universidad Nacional Autónoma de México.

Familiar-López M (2007) La variacion de la temperatura diaria y su relacion con los brotes de quitridiomicosis en anfibios de las montañas de Guerrero y Oaxaca, Mexico. Thesis, Facultad de Ciencias, Universidad Nacional Autónoma de México.

Frías-Alvarez P, Vredenburg VT, Familiar-López M, Longcore JE, González-Bernal E, Santos-Barrera G, Zambrano L, Parra-Olea G (2008) Chytridiomycosis Survey in Wild and Captive Mexican Amphibians. EcoHealth 5:18–26. DOI: 10.1007/s10393-008-0155-3 [Online February 20, 2008]

García-Feria LM, Brousset DM, Cervantes-Olivares RA (2019) Determinant abiotic and biotic factors for the presence of Batrachochytrium dendrobatidis in Mexican amphibians. Acta zoológica Mexicana 35:1–18. DOI: 10.21829/azm.2019.3502066 [Online December 29, 2019]

Hernández-Martínez LA, Romero-Méndez U, González-Barrios JL, García-De la Peña MC, Amézquita-Torres A (2019) Nuevos registros y prevalencia de *Batrachochytrium dendrobatidis* en anuros de la cuenca Nazas-Aguanaval en la región norte-centro de México. Revista Mexicana de Biodiversidad 90:1–9. DOI: 10.22201/ib.20078706e.2019.90.2934 [Online March 04, 2019]

Jacinto-Maldonado M, García-Peña GE, Paredes-León R, Saucedo B, Sarmiento-Silva RE, García A, Martínez-Gómez D, Ojeda M, Del Callejo E, Suzán, G (2019) Chiggers (Acariformes: Trombiculoidea) do not increase rates of infection by *Batrachochytrium dendrobatidis* fungus in the endemic Dwarf Mexican Treefrog Tlalocohyla smithii (Anura: Hylidae). International journal for parasitology. Parasites and wildlife 11:163–173. DOI: 10.1016/j.ijppaw.2019.12.005 [Online December 16, 2019]

Jacinto-Maldonado M, Lesbarrères L, Rebollar EA, Delia-Basanta M, González-Grijalva B, Robles-Morúa A, Álvarez-Bajo O, Vizuete-Jaramillo E, Paredes-León R, Meza-Figueroa D (2024) Batrachochytrium dendrobatidis

and Hannemania mite's relationships with Mexican amphibians in disturbed environments. Frontiers in Amphibian and Reptile Science 2: 1372993 DOI: 10.3389/famrs.2024.1372993 [Online April 03, 2024]

Leyte-Manrique A, Alejo-Iturvide F, Aguillón-Gutiérrez DR, Ochoa-Ochoa L, Rodríguez-Gutiérrez M.F (2023) Pathogens in anurans from a seasonal tropical environment in Guanajuato, Mexico. Animal Biodiversity and Conservation 46:35-46 DOI: 10.32800/abc.2023.46.0035 [Online December 19, 2022]

Lips KR, Mendelson III JR, Muñoz-Alonso A, Canseco-Márquez L, Mulcahy DG (2004) Amphibian population declines in montane southern Mexico: resurveys of historical localities. Biological Conservation 119:555–564 DOI: 10.1016/j.biocon.2004.01.017 [Online January 14, 2004]

Luja VH, Rodríguez-Estrella R, Ratzlaff K, Parra-Olea G, Ramírez-Bautista A (2012) The Chytrid Fungus *Batrachochytrium dendrobatidis* in Isolated Populations of the Baja California Treefrog *Pseudacris hypochondriaca curta* in Baja California Sur, Mexico. The Southwestern Naturalist 57:323–327. DOI: 10.1894/0038-4909-57.3.323 [Online September 01, 2012]

Luría-Manzano R, Canseco-Márquez A, Frías-Alvarez P (2011) *Batrachochytrium dendrobatidis* in *Plectrohyla arborescandens* (Anura: Hylidae) Larvae at a Montane Site in the Sierra Negra, Puebla, México. Herpetological Review 42:552–554.

Méndoza-Almeralla C, López-Velázquez A, Longo AV, Parra-Olea G (2016) Temperature treatments boost subclinical infections of *Batrachochytrium dendrobatidis* in a Mexican salamander (*Pseudoeurycea leprosa*). Revista Mexicana de Biodiversidad 87:171–179 DOI: 10.1016/j.rmb.2016.01.020 [Online February 28, 2016]

Méndoza-Almeralla C, Tafolla-Venegas D, González-Pardo C, Suazo-Ortuño I (2023) Primer Registro de Infección por *Batrachochytrium dendrobatidis* y por el Nematodo del Género *Capillaria* y la Ausencia de Infección por *Ribeiroia ondatrae* en *Ambystoma ordinarium*. Revista Latinoamericana de Herpetología 6:5–13. DOI: 0.22201/fc.25942158e.2023.4.615 [Online October 09, 2023]

Murrieta-Galindo R, Parra-Olea G, González-Romero A, López-Barrera F, Vredenburg VT (2014) Detection of *Batrachochytrium dendrobatidis* in amphibians inhabiting cloud forests and coffee agroecosystems in central Veracruz, Mexico. European Journal of Wildlife Research, 60:431–439. DOI: 10.1007/s10344-014-0800-9 [Online February 18, 2014]

Nava-González B, Suazo-Ortuño I, López PB, Maldonado-López Y, Lopez-Toledo L, Raggi L, Parra-Olea G, Alvarado-Díaz J, Gómez-Gil B (2021) Inhibition of *Batrachochytrium dendrobatidis* Infection by Skin Bacterial Communities in Wild Amphibian Populations. Microbial Ecology 82:666–676. DOI: 10.1007/s00248-021-01706-x [Online February 18, 2021]

Nava-González BA, Suazo-Ortuño I, Parra-Olea G, López-Toledo L, Alvarado-Díaz J (2020) *Batrachochytrium dendrobatidis* infection in amphibians from a high elevation habitat in the trans-Mexican volcanic belt. Aquatic Ecology 54:75–87. DOI: 10.1007/s10452-019-09727-y [Online September 26, 2019]

Peralta-García A, Adams AJ, Briggs CJ, Galina-Tessaro P, Valdez-Villavicencio JH, Hollingsworth BD, Bradley Shaffer H, Fisher RN (2018) Occurrence of *Batrachochytrium dendrobatidis* in anurans of the Mediterranean region of Baja California, México. Disease of Aquatic Organisms 127:193–200. DOI: 10.3354/dao03202 [Online March 08, 2018]

Quintero-Díaz GE, Muñoz-Alonso LA, Lips KR (2013) *Batrachochytrium dendrobatidis*: un hongo patógeno de anfibios. In: La biodiversidad en Chiapas: Estudio de Estado, Cruz-Angón A, Melgarejo ED, Camacho-Rico F, Nájera-Cordero KC (editors), tuxtla Gutiérrez, Chiapas: Comisión Nacional para el Conocimiento y Uso de la Biodiversidad, pp 361–364

Van Rooij P, Martel A, Nerz J, Voitel S, Van Immerseel F, Haesebrouck F, Pasmans F (2011) Detection of *Batrachochytrium dendrobatidis* in Mexican Bolitoglossine Salamanders Using an Optimal Sampling Protocol. EcoHealth 8:237–243. DOI: 10.1007/s10393-011-0704-z [Online September 13, 2011]

Table S2. Ecophysiological suitability index for *Batrachochytrium dendrobatidis* in the extension of 203 federal natural protected areas in Mexico. Area represents only the terrestrial surface.

Natural protected area	State	Area (Ha)	mean	σ	min	max
Cumbres del Ajusco	Ciudad de México	920	1.00	0.00	1.00	1.00
Nevado de Toluca	Estado de México	53591	1.00	0.03	0.63	1.00
Iztaccíhuatl-Popocatépetl	Estado de México, Puebla and Morelos	39819	1.00	0.02	0.63	1.00
Cofre de Perote o Nauhcampatépetl	Veracruz	11531	1.00	0.00	1.00	1.00
Pico de Orizaba	Veracruz and Puebla	19750	1.00	0.00	1.00	1.00
Arrecife Alacranes	Yucatán	53	1.00	0.00	1.00	1.00
Constitución de 1857	Baja California	5009	0.99	0.06	0.63	1.00
Desierto de los Leones	Ciudad de México	1529	0.92	0.14	0.63	1.00
Volcán Nevado de Colima	Jalisco and Colima	6555	0.91	0.13	0.63	1.00
Mariposa Monarca	Michoacán and Estado de México	56259	0.90	0.15	0.63	1.00
Sierra de San Pedro Mártir	Baja California	72911	0.89	0.19	0.41	1.00
Banco Chinchorro	Quintana Roo	586	0.89	0.30	0.06	1.00
Barranca del Cupatitzio	Michoacán	458	0.88	0.03	0.86	0.91
La Montaña Malinche o Matlalcuéyatl	Tlaxcala and Puebla	46112	0.87	0.16	0.63	1.00
Insurgente Miguel Hidalgo and Costilla	Estado de México and Ciudad de México	1890	0.86	0.18	0.63	1.00
Bosencheve	Estado de México and Michoacán	14600	0.85	0.18	0.63	1.00
Papigochic	Chihuahua	222764	0.84	0.18	0.54	1.00
Cerro Mohinora	Chihuahua	9126	0.84	0.17	0.63	1.00
Peña Colorada	Querétaro	4844	0.82	0.11	0.47	0.91
El Chico	Hidalgo	2739	0.81	0.17	0.63	1.00
Isla Guadalupe	Baja California	26277	0.80	0.13	0.49	0.91
Lagunas de Zempoala	Morelos and Estado de México	4790	0.78	0.18	0.63	1.00
Benito Juárez	Oaxaca	2592	0.78	0.08	0.63	0.91
Pico de Tancítaro	Michoacán	23406	0.77	0.11	0.63	1.00
Fuentes Brotantes de Tlalpan	Ciudad de México	129	0.76	0.00	0.76	0.76
Lomas de Padierna	Ciudad de México	1161	0.76	0.00	0.76	0.76
Ciénegas del Lerma	Estado de México	3024	0.76	0.00	0.76	0.76
Los Remedios	Estado de México	400	0.76	0.00	0.76	0.76
Sacromonte	Estado de México	44	0.76	0.00	0.76	0.76
Z.P.F.V. la Cuenca Hidrográfica del Río Necaxa	Hidalgo and Puebla	42129	0.76	0.11	0.21	1.00
El Cimatario	Querétaro	2448	0.76	0.10	0.70	0.91
El Potosí	San Luis Potosí	2000	0.76	0.09	0.70	0.91
Xicoténcatl	Tlaxcala	851	0.76	0.00	0.76	0.76
Sierra de órganos	Zacatecas	1125	0.76	0.00	0.76	0.76
Campo Verde	Chihuahua and Sonora	108067	0.75	0.19	0.54	1.00

Desierto del Carmen o de Nixcongo	Estado de México	529	0.75	0.02	0.70	0.76
Molino de Flores NetzahualcÃ³yotl	Estado de México	46	0.75	0.03	0.70	0.76
Cerro de Garnica	Michoacán	1936	0.75	0.13	0.63	1.00
Sierra de Álvarez	San Luis Potosí	16900	0.75	0.06	0.70	0.91
Sierra de San Miguelito	San Luis Potosí	111160	0.75	0.08	0.47	0.91
C.A.D.N.R. 001 Pabellón	Aguascalientes and Zacatecas	97700	0.74	0.04	0.63	1.00
Volcán Tacaná	Chiapas	6378	0.74	0.17	0.15	1.00
La Michilía	Durango	35000	0.74	0.05	0.47	1.00
San Quintín	Baja California	86	0.73	0.03	0.70	0.76
Cerro de La Estrella	Ciudad de México	1183	0.73	0.07	0.70	0.91
Z.P.F.T.C.C. de los ríos Valle de Bravo, Malacatepec, Tilostoc and Temascaltepec	Estado de México	140234	0.73	0.19	0.05	1.00
Los Mármoles	Hidalgo	23150	0.73	0.27	0.05	0.92
Cumbres de Majalca	Chihuahua	4701	0.72	0.09	0.54	0.76
C.A.D.N.R. 026 Bajo Río San Juan	Coahuila and Nuevo León	197157	0.72	0.22	0.05	1.00
Lago de Texcoco	Estado de México	14000	0.71	0.04	0.70	0.91
El Histórico Coyoacán	Ciudad de México	40	0.70	0.00	0.70	0.70
El Tepeyac	Ciudad de México and Estado de México	1500	0.70	0.00	0.70	0.70
Tula	Hidalgo	100	0.70	0.00	0.70	0.70
Insurgente José María Morelos	Michoacán	7192	0.70	0.21	0.05	0.91
Corredor Biológico Chichinautzin	Ciudad de México, Morelos and Estado de México	37302	0.69	0.21	0.05	1.00
Sierra de Quila	Jalisco	15193	0.69	0.22	0.05	0.92
Cañón del Río Blanco	Veracruz and Puebla	48800	0.66	0.32	0.05	1.00
Cascada de Bassaseachic	Chihuahua	5803	0.65	0.13	0.41	0.76
Sierra La Mojonera	San Luis Potosí and Zacatecas	9202	0.65	0.11	0.47	0.76
Tutuaca	Chihuahua and Sonora	436986	0.64	0.17	0.05	1.00
Rayón	Michoacán	25	0.63	0.00	0.63	0.63
Complejo Lagunar Ojo de Liebre	Baja California and Baja California Sur	79329	0.61	0.34	0.05	1.00
Gogorrón	San Luis Potosí	36500	0.61	0.21	0.41	0.92
Cumbres de Monterrey	Nuevo León and Coahuila	177396	0.60	0.32	0.05	1.00
Playa Ceuta	Sinaloa	503	0.59	0.41	0.19	1.00
Zona marina del Archipiélago de Espíritu Santo	Baja California Sur	0	0.58	0.42	0.15	1.00
El Triunfo	Chiapas	119177	0.58	0.24	0.00	0.91
Janos	Chihuahua	526482	0.56	0.12	0.48	1.00
Playa Huizache Caimanero	Sinaloa	451	0.55	0.40	0.19	1.00
Arrecife de Puerto Morelos	Quintana Roo	38	0.53	0.47	0.06	1.00

El Jabalí	Colima	5179	0.52	0.34	0.05	0.91
Médanos de Samalayuca	Chihuahua	56134	0.50	0.02	0.47	0.54
El Tepozteco	Morelos and Ciudad de México	23259	0.50	0.33	0.05	1.00
Lagunas de Montebello	Chiapas	6546	0.49	0.00	0.49	0.49
La Primavera	Jalisco	30500	0.48	0.36	0.05	0.91
Playa Puerto Arista	Chiapas	727	0.47	0.44	0.11	1.00
C.A.D.N.R. 043 Estado de Nayarit	Aguascalientes, Jalisco, Durango, Nayarit and Zacatecas	2329027	0.46	0.32	0.00	1.00
Sierra de Manantlán	Jalisco and Colima	139577	0.46	0.32	0.00	0.92
Sierra Gorda de Guanajuato	Guanajuato and Querétaro	236883	0.43	0.35	0.05	0.92
Playa Río Lagartos	Yucatán	827	0.43	0.49	0.00	1.00
Playa Piedra de Tlacoyunque	Guerrero	98	0.41	0.42	0.11	1.00
Lago de Camécuaro	Michoacán	6	0.41	0.00	0.41	0.41
Cerro de Las Campanas	Querétaro	58	0.41	0.00	0.41	0.41
Sierra La Laguna	Baja California Sur	112437	0.40	0.36	0.05	0.91
Tehuacán-Cuicatlán	Puebla and Oaxaca	490187	0.40	0.33	0.00	1.00
Barranca de Metztitlán	Hidalgo	96043	0.38	0.37	0.05	0.91
Bavispe	Sonora	200901	0.37	0.23	0.05	1.00
Maderas del Carmen	Coahuila	208381	0.36	0.26	0.05	1.00
Sierra Gorda	Querétaro, Guanajuato, San Luis Potosí e Hidalgo	383567	0.36	0.33	0.05	0.92
Playa Mismaloya	Jalisco	811	0.33	0.47	0.00	1.00
Islas del Pacífico de la Península de Baja California	Baja California and Baja California Sur	70140	0.32	0.29	0.05	1.00
Z.P.F. en los terrenos que se encuentran en los mpios. de La Concordia, Ángel Albino Corzo, Villa Flores and Jiquipilas	Chiapas	177546	0.30	0.21	0.13	0.91
Islas Marías	Nayarit	24295	0.30	0.26	0.15	1.00
Valle de los Cirios	Baja California	2521988	0.29	0.24	0.05	1.00
Revillagigedo	S/E	15518	0.27	0.26	0.00	0.82
Playa El Tecuán	Jalisco	52	0.25	0.35	0.00	1.00
La Sepultura	Chiapas	167310	0.24	0.22	0.00	0.91
C.A.D.N.R. 004 Don Martín	Coahuila	1519385	0.24	0.25	0.05	0.92
Zona marina Bahía de los Ángeles, canales de Ballenas and de Salsipuedes	Baja California	483	0.22	0.15	0.05	0.49
Nahá	Chiapas	3847	0.22	0.10	0.15	0.65
Playa Rancho Nuevo	Tamaulipas	1844	0.22	0.22	0.15	1.00
Los Novillos	Coahuila	38	0.21	0.00	0.21	0.21
Laguna Madre and Delta del Río Bravo	Tamaulipas	572809	0.20	0.20	0.15	1.00
Playa Chacahua	Oaxaca	546	0.19	0.33	0.06	1.00

Los Tuxtlas	Veracruz	155122	0.19	0.20	0.00	1.00
Selva El Ocote	Chiapas	101288	0.18	0.10	0.00	0.65
Cuatro Ciénegas	Coahuila	84347	0.18	0.07	0.05	0.47
Meseta de Cacaxtla	Sinaloa	50862	0.18	0.05	0.00	1.00
Islas del Golfo de California	B.C., B.C.S., Sonora and Sinaloa	374554	0.17	0.15	0.05	1.00
Sierra de Tamaulipas	Tamaulipas	308888	0.17	0.03	0.05	0.49
Alto Golfo de California and Delta del Río Colorado	Baja California and Sonora	407148	0.16	0.05	0.05	1.00
Bahía de Loreto	Baja California Sur	21692	0.16	0.05	0.15	1.00
Cañón del Sumidero	Chiapas	21789	0.16	0.07	0.00	0.49
Cañón de Santa Elena	Chihuahua	277210	0.16	0.17	0.05	0.76
General Juan Álvarez	Guerrero	528	0.16	0.19	0.05	0.49
Grutas de Cacahuamilpa	Guerrero	1600	0.16	0.02	0.15	0.21
El Pinacate and Gran Desierto de Altar	Sonora	714557	0.16	0.01	0.05	0.21
Balandra	Baja California Sur	1320	0.15	0.00	0.15	0.15
Cabo Pulmo	Baja California Sur	39	0.15	0.00	0.15	0.15
Cabo San Lucas	Baja California Sur	208	0.15	0.00	0.15	0.15
Loreto II	Baja California Sur	6218	0.15	0.05	0.05	0.21
NopolÃ³	Baja California Sur	2077	0.15	0.00	0.15	0.15
Bonampak	Chiapas	4357	0.15	0.07	0.00	0.19
Metzabok	Chiapas	3368	0.15	0.00	0.15	0.15
El Sabinal	Nuevo León	8	0.15	0.00	0.15	0.15
Sierra del Abra Tanchipa	San Luis Potosí and Tamaulipas	21464	0.15	0.01	0.13	0.19
Sierra de Álamos-Río Cuchujaqui	Sonora, Sinaloa and Chihuahua	92890	0.15	0.05	0.05	0.41
Sierra de Huautla	Morelos, Puebla and Guerrero	59031	0.14	0.07	0.00	0.86
Boquerón de Tonalá	Oaxaca	3912	0.14	0.10	0.05	0.49
Sistema Arrecifal Veracruzano	Veracruz	12	0.14	0.26	0.00	1.00
El Vizcaíno	Baja California and Baja California Sur	2259003	0.13	0.16	0.05	1.00
La Encrucijada	Chiapas	115653	0.12	0.09	0.11	1.00
Playa Tierra Colorada	Guerrero	264	0.12	0.19	0.06	1.00
Montes Azules	Chiapas	331200	0.11	0.11	0.00	0.65
Río Bravo del Norte	Chihuahua and Coahuila	2175	0.11	0.08	0.05	0.21
Cerro de la Silla	Nuevo León	6039	0.11	0.16	0.05	0.86
Ricardo Flores Magón	Oaxaca	1813	0.11	0.00	0.11	0.11
Cascada de Agua Azul	Chiapas	2580	0.10	0.09	0.00	0.19
Vicente Guerrero	Guerrero	724	0.10	0.02	0.06	0.11
Huatulco	Oaxaca	6375	0.10	0.02	0.06	0.11
Ocampo	Coahuila and Chihuahua	344238	0.09	0.08	0.05	0.76
Huatulco II	Oaxaca	2238	0.09	0.03	0.06	0.11
Zicuirán -Infiernillo	Michoacán	265118	0.08	0.07	0.00	0.21

Playa Mexiquillo	Michoacán	100	0.08	0.03	0.06	0.11
Yagul	Oaxaca	1076	0.08	0.07	0.05	0.21
La porción norte and la franja costera oriental, terrestres and marinas de la Isla de Cozumel	Quintana Roo	5733	0.08	0.16	0.06	1.00
Juan M. Banderas	Sinaloa	2489	0.08	0.09	0.00	0.19
Río Lagartos	Yucatán and Quintana Roo	60348	0.08	0.27	0.00	1.00
Laguna de Términos	Campeche and Tabasco	547279	0.07	0.11	0.06	1.00
Río Celestún	Campeche and Yucatán	61927	0.07	0.12	0.06	1.00
Hermenegildo Galeana	Guerrero	282	0.07	0.03	0.06	0.11
Los Petenes	Campeche	100867	0.06	0.00	0.06	0.06
Playa Colola	Michoacán	77	0.06	0.00	0.06	0.06
Playa Maruata	Michoacán	12	0.06	0.00	0.06	0.06
Lagunas de Chacahua	Oaxaca	14896	0.06	0.08	0.06	1.00
Playa Escobilla	Oaxaca	263	0.06	0.00	0.06	0.06
Bajos de Coyula	Oaxaca	1923	0.06	0.02	0.06	0.11
Costa Occ. de I. Mujeres, Pta. Cancún and Pta. Nizuc	Quintana Roo	1	0.06	0.00	0.06	0.06
Isla Contoy	Quintana Roo	230	0.06	0.00	0.06	0.06
Manglares de Nichupté	Quintana Roo	4257	0.06	0.00	0.06	0.06
Arrecifes de Sian Ka'an	Quintana Roo	1361	0.06	0.00	0.06	0.06
Sian Ka'an	Quintana Roo	375012	0.06	0.04	0.00	1.00
Uaymil	Quintana Roo	89118	0.06	0.00	0.00	0.06
Arrecifes de Xcalak	Quintana Roo	4522	0.06	0.01	0.06	0.11
Arrecifes de Cozumel	Quintana Roo	82	0.06	0.00	0.06	0.06
Playas de Isla Contoy	Quintana Roo	11	0.06	0.00	0.06	0.06
Jacinto Pat	Quintana Roo	17	0.06	0.00	0.06	0.06
Cenote Aerolito	Quintana Roo	10	0.06	0.00	0.06	0.06
Playa Delfines	Quintana Roo	5	0.06	0.00	0.06	0.06
San Buenaventura	Quintana Roo	38	0.06	0.00	0.06	0.06
Wanha'	Tabasco	38256	0.06	0.00	0.06	0.06
Pantanos de Centla	Tabasco and Campeche	302707	0.06	0.00	0.06	0.06
Calakmul	Campeche	728909	0.05	0.08	0.00	0.19
Palenque	Chiapas	1772	0.05	0.05	0.00	0.19
Mapimí	Durango, Chihuahua and Coahuila	342388	0.05	0.03	0.05	0.41
Chamela-Cuixmala	Jalisco	13142	0.05	0.08	0.00	0.19
Isla San Pedro Mártir	Sonora	127	0.05	0.00	0.05	0.05
El Veladero	Guerrero	3617	0.04	0.05	0.00	0.19
Lacan-Tun	Chiapas	61874	0.03	0.07	0.00	0.19
Cañón del Usumacinta	Tabasco	46128	0.03	0.06	0.00	0.19
Balam Kú	Campeche	463442	0.02	0.03	0.00	0.19
Balam Kin	Campeche	115658	0.02	0.05	0.00	0.19
Caribe Mexicano	Quintana Roo	28589	0.02	0.15	0.00	1.00

Yaxchilán	Chiapas	2621	0.01	0.03	0.00	0.19
Marismas Nacionales Nayarit	Nayarit	133854	0.01	0.05	0.00	1.00
Yum Balam	Quintana Roo	52308	0.01	0.12	0.00	1.00
Chan-Kin	Chiapas	12185	0.00	0.00	0.00	0.00
Las Huertas	Colima	167	0.00	0.00	0.00	0.00
Islas La Pajarera, Cocinas, Mamut, Colorada, San Pedro, San Agustín, San Andrés and Negrita and los Islotes Los Anegados, Novillas, Mosca and Submarino	Jalisco	1981	0.00	0.00	0.00	0.00
Playa Teopa	Jalisco	31	0.00	0.00	0.00	0.00
Playa Cuitzmala	Jalisco	21	0.00	0.00	0.00	0.00
Otoch Ma'ax Yetel Kooh	Quintana Roo	5367	0.00	0.00	0.00	0.00
Jaguar	Quintana Roo	2250	0.00	0.00	0.00	0.00
Tulum	Quintana Roo	664	0.00	0.00	0.00	0.00
Bala'an K'aax	Quintana Roo, Yucatán and Campeche	128390	0.00	0.00	0.00	0.00
Dzibilchantún	Yucatán	539	0.00	0.00	0.00	0.00
Zona marina del Archipiélago de San Lorenzo	Baja California	0	-	-	-	-
Isla Isabel	Nayarit	194	0.00	0.00	0.00	0.00
Islas Marietas	Nayarit	71	0.00	0.00	0.00	0.00
Pacifico Mexicano Profundo	Nayarit, Jalisco, Colima, Michoacán, Guerrero, Oaxaca and Chiapas	0	-	-	-	-
TiburÃ³n Ballena	Quintana Roo	0	-	-	-	-
Ventilas Hidrotermales de la Cuenca de Guaymas and de la Dorsal del Pacifico Oriental	Sinaloa	0	-	-	-	-
Sistema Arrecifal Lobos-Tuxpan	Veracruz	0	-	-	-	-

Table S3. Ecophysiological suitability index for *Batrachochytrium dendrobatidis* in the extension of 299 statal natural protected areas in Mexico.

Natural protected area	State	Area (Ha)	mean	σ	min	max
Centro Ceremonial Mazahua	Estado de México	19	1.00	0.00	1.00	1.00
Quebrada de Santa Bárbara	Durango	65	1.00	0.00	1.00	1.00
San Pedro en el Monte	Veracruz	441	1.00	0.00	1.00	1.00
Cerro El Potosí	Nuevo León	971	1.00	0.00	1.00	1.00
Santuario del Agua Presa Brockman y Victoria	Estado de México	1555	1.00	0.00	1.00	1.00
Santuario del Agua Presa Corral de Piedra	Estado de México	3365	0.98	0.08	0.63	1.00
Lic. Isidro Fabela	Estado de México	2847	0.97	0.10	0.63	1.00
San Juan del Monte	Veracruz	602	0.94	0.14	0.63	1.00
Cerrito de la Independencia	Michoacán	3	0.91	0.00	0.91	0.91
Megaparque	Guanajuato	14	0.91	0.00	0.91	0.91
Cerro Punhuato	Michoacán	78	0.91	0.00	0.91	0.91
Lic. Salvador Bernal Murguía (sector El Madroño)	Michoacán	7	0.91	0.00	0.91	0.91
Lic. Salvador Bernal Murguía (sector El Charco)	Michoacán	10	0.91	0.00	0.91	0.91
Cerro Punhuato (ampliación)	Michoacán	40	0.91	0.00	0.91	0.91
La Martinica	Veracruz	119	0.91	0.00	0.91	0.91
Paseo de la Presa	San Luis Potosí	342	0.91	0.00	0.91	0.91
Cerro Cualtenco	Estado de México	364	0.91	0.00	0.91	0.91
La Mesa de Tzitzio	Michoacán	77	0.90	0.02	0.86	0.91
Santuario del Agua y Forestal Manantiales Cascada Diamantes	Estado de México	7023	0.88	0.14	0.63	1.00
Zempoala - La Bufa "Otomí-Mexica"	Estado de México	71681	0.88	0.16	0.63	1.00
Santuario del Agua y Forestal Manantial El Salto de Atlautla - Ecatzingo	Estado de México	9038	0.87	0.15	0.63	1.00
Molino de San Roque	Veracruz	17	0.86	0.00	0.86	0.86
Cerro Macuiltepetl	Veracruz	29	0.86	0.00	0.86	0.86
Cerro La Galaxia	Veracruz	33	0.86	0.00	0.86	0.86
Bosque Mesófilo Nevado de Colima (sector: Barranca de Atenquique-Loma Alta)	Jalisco	1671	0.85	0.13	0.63	1.00
Santuario del Agua y Forestal Subcuenca Tributaria Río Mayorazgo-Temoaya	Estado de México	25080	0.84	0.16	0.63	1.00
San Miguel Topilejo	Distrito Federal	5831	0.83	0.18	0.63	1.00
San Nicolás Totolapan	Distrito Federal	1975	0.83	0.15	0.63	1.00
Bosque Mesófilo Nevado de Colima (sector: Barranca de Alseseca)	Jalisco	2433	0.81	0.11	0.63	1.00
Bosque Mesófilo Nevado de Colima (sector: El Borboyón)	Jalisco	1627	0.81	0.13	0.49	0.91
Sistema Tetzcotzingo	Estado de México	7771	0.80	0.12	0.63	1.00
El Tangano	Queretaro	848	0.78	0.10	0.70	0.91
Las Tinajas de Huandacareo	Michoacán	252	0.78	0.10	0.70	0.92
Cañón Pino del Campo	Nuevo León	2527	0.77	0.10	0.70	0.91

La Purísima (Bosque de Oyamel)	Nuevo León	835	0.76	0.05	0.70	0.91
Tenancingo - Malinalco - Zumpahuacan	Estado de México	33135	0.76	0.09	0.49	0.91
Santuario del Agua Valle de Bravo	Estado de México	15199	0.76	0.05	0.70	0.91
Pinal del Zamorano	Guanajuato	13317	0.76	0.06	0.70	0.91
Jose María Velasco	Estado de México	4	0.76	0.00	0.76	0.76
Sierra de Guadalupe / La Armella	Edo. México - DF	4	0.76	0.00	0.76	0.76
La Cañada	Estado de México	5	0.76	0.00	0.76	0.76
Tiacaque	Estado de México	7	0.76	0.00	0.76	0.76
Cerro La Paila-El Susto	Hidalgo	12	0.76	0.00	0.76	0.76
Las Lajas - Cerro del Lobo	Hidalgo	22	0.76	0.00	0.76	0.76
Cerro La Paila - Matías Rodríguez	Hidalgo	24	0.76	0.00	0.76	0.76
La Ciénega	Tlaxcala	42	0.76	0.00	0.76	0.76
Rancho Teometitla	Tlaxcala	48	0.76	0.00	0.76	0.76
Pancho Poza	Veracruz	57	0.76	0.00	0.76	0.76
Rancho Los Pitzocales o El Carmen	Tlaxcala	63	0.76	0.00	0.76	0.76
Barranca Río La Pastora, R. La Loma y R. San Joaquín (La Pastora)	Estado de México	86	0.76	0.00	0.76	0.76
Cubitos	Hidalgo	90	0.76	0.00	0.76	0.76
Huitepec - Los Alcanfores	Chiapas	103	0.76	0.00	0.76	0.76
Ecoguardas	Distrito Federal	132	0.76	0.00	0.76	0.76
Alameda Poniente San José de la Pila	Estado de México	177	0.76	0.00	0.76	0.76
La Armella	Distrito Federal	189	0.76	0.00	0.76	0.76
Espíritu Santo (Cerro de Chiluca)	Estado de México	233	0.76	0.00	0.76	0.76
Atizapan - Valle Escondido (Los Ciervos)	Estado de México	307	0.76	0.00	0.76	0.76
Mario Molina Pasquel / Pinal del Zamorano	Queret. y Guanaj.	309	0.76	0.00	0.76	0.76
Hermenegildo Galeana	Estado de México	335	0.76	0.00	0.76	0.76
Parque Ecológico de la Ciudad de México	Distrito Federal	716	0.76	0.00	0.76	0.76
Sierra Morelos	Estado de México	1188	0.76	0.00	0.76	0.76
Mario Molina Pasquel	Queretaro	1281	0.76	0.00	0.76	0.76
Cerro Gordo	Estado de México	3034	0.76	0.00	0.76	0.76
Rancho Nuevo	Chiapas	1698	0.76	0.00	0.76	0.76
Sierra de Tepozotlán	Estado de México	9232	0.76	0.00	0.76	0.76
Bosque de Tlalpan	Distrito Federal	251	0.76	0.00	0.76	0.76
Sierra Patlachique	Estado de México	3082	0.76	0.00	0.76	0.76
Barranca Río La Pastora, R. La Loma y R. San Joaquín (La Loma)	Estado de México	11	0.76	0.00	0.76	0.76
Santa Marta de Abajo	Nuevo León	21	0.76	0.02	0.70	0.76
Barrancas Huizachal, Arroyo Santa Cruz y A. Plan de Zanja (Plan de la Zanja)	Estado de México	17	0.76	0.00	0.76	0.76
Barranca México 68	Estado de México	4	0.76	0.00	0.76	0.76
Barranca de Tecamachalco	Estado de México	6	0.76	0.00	0.76	0.76
Barrancas Huizachal, Arroyo Santa Cruz y A. Plan de Zanja (Ampl. Santa Cruz)	Estado de México	9	0.76	0.00	0.76	0.76
Barranca de Tecamachalco	Estado de México	9	0.76	0.00	0.76	0.76

Barrancas Huizachal, Arroyo Santa Cruz y A. Plan de Zanja (Santa Cruz)	Estado de México	11	0.76	0.00	0.76	0.76
Barranca Río La Pastora, R. La Loma y R. San Joaquín (Río San Joaquín)	Estado de México	22	0.76	0.00	0.76	0.76
Bosques de las Lomas	Distrito Federal	29	0.76	0.00	0.76	0.76
Parque Ecológico Público Diego Muñóz Camargo	Tlaxcala	30	0.76	0.00	0.76	0.76
Barrancas Huizachal, Arroyo Santa Cruz y A. Plan de Zanja (El Huizachal)	Estado de México	32	0.76	0.00	0.76	0.76
Metropolitano de Naucalpan	Estado de México	35	0.76	0.00	0.76	0.76
El Recreo	Chiapas	45	0.76	0.00	0.76	0.76
Ecológico de Uruapan	Michoacán	52	0.76	0.00	0.76	0.76
Gertrude Duby	Chiapas	65	0.76	0.00	0.76	0.76
Tercera Sección del Bosque de Chapultepec II	Distrito Federal	92	0.76	0.00	0.76	0.76
Humedales de Montaña La Kisst	Chiapas	111	0.76	0.00	0.76	0.76
Humedales de Montaña María Eugenia	Chiapas	116	0.76	0.00	0.76	0.76
Cerro Colorado	Estado de México	121	0.76	0.00	0.76	0.76
Tercera Sección del Bosque de Chapultepec I	Distrito Federal	157	0.76	0.00	0.76	0.76
Sierra Hermosa	Estado de México	507	0.76	0.00	0.76	0.76
Santuario del Agua y Forestal Presa Guadalupe	Estado de México	1787	0.76	0.00	0.76	0.76
La Trinidad (2002)	Nuevo León	3250	0.76	0.00	0.76	0.76
Sierra de Guadalupe	Edo. México - DF	7698	0.76	0.00	0.76	0.76
Santuario del Agua y Forestal Presa Taxhimay	Estado de México	8208	0.76	0.02	0.63	0.76
Cuenca de la Esperanza	Guanajuato	1814	0.76	0.01	0.70	0.76
Santuario del Agua Laguna de Zumpango	Estado de México	19985	0.76	0.01	0.70	0.76
La Purisíma (Bosque de Cedro)	Nuevo León	17	0.76	0.03	0.70	0.76
Bosque Mesófilo Nevado de Colima (sector: Barranca de los Bueyes)	Jalisco	1412	0.75	0.05	0.63	0.91
Francisco Javier Clavijero	Veracruz	80	0.75	0.16	0.49	0.86
Sierra de Lobos	Guanajuato	102451	0.75	0.05	0.41	0.91
Santuario del Agua Presa Ñado	Estado de México	4285	0.75	0.03	0.63	0.76
Chanal	Chiapas	4261	0.75	0.02	0.70	0.76
Santuario del Agua Sistema Hidrológico Presa Huapango	Estado de México	70553	0.75	0.04	0.63	1.00
Santuario del Agua y Forestal Subcuenca Tributaria Río San Lorenzo	Estado de México	12596	0.75	0.11	0.63	1.00
Cuenca de la Soledad	Guanajuato	2757	0.74	0.02	0.70	0.76
Peña Alta	Guanajuato	11364	0.74	0.02	0.70	0.76
Serranía de Zapalinamé	Coahuila	25125	0.74	0.05	0.63	1.00
Nahuatlaca - Matlazinca	Estado de México	24306	0.74	0.11	0.05	1.00
Sierra de los Agustinos	Guanajuato	19022	0.73	0.11	0.41	0.91
Sierra Fría	Aguascalientes	106030	0.73	0.07	0.41	1.00
Santuario del Agua y Forestal Subcuenca Tributaria Arroyo Sila	Estado de México	53186	0.73	0.12	0.63	1.00
Monte Alto	Estado de México	619	0.73	0.03	0.70	0.76

Sandía El Grande	Nuevo León	1862	0.73	0.19	0.41	0.91
Región denominada Cerro Blanco RB La Michilía	Durango	5406	0.73	0.06	0.63	1.00
Tzama Cum Pumy	Chiapas	102	0.72	0.08	0.70	0.91
Ejidos de Xochimilco y San Gregorio Atlapulco	Distrito Federal	2507	0.72	0.03	0.70	0.76
Santuario del Agua y Forestal Subcuenca Tributaria Presa Antonio Alzate	Estado de México	11480	0.72	0.08	0.63	1.00
El Oso Bueno	Estado de México	15072	0.72	0.13	0.63	1.00
Santuario del Agua y Forestal Presa Villa Victoria	Estado de México	46271	0.72	0.15	0.63	1.00
Tollocan - Calimaya (Zoológico de Zacango)	Estado de México	160	0.72	0.06	0.63	0.76
Cerro de los Amoles	Guanajuato	6932	0.71	0.10	0.47	0.91
Ecológico de Capácuaro	Michoacán	8	0.70	0.00	0.70	0.70
San José Chalco	Estado de México	17	0.70	0.00	0.70	0.70
Cerro Pelón	Michoacán	24	0.70	0.00	0.70	0.70
Parque Estado de México - Naucalli	Estado de México	52	0.70	0.00	0.70	0.70
Santuario del Agua Lagunas de Xico	Estado de México	121	0.70	0.00	0.70	0.70
Sierra de Santa Catarina II	Distrito Federal	220	0.70	0.00	0.70	0.70
Ex-Escuela Central Agrícola de la Huerta	Michoacán	269	0.70	0.00	0.70	0.70
Cerro del Estribo Grande	Michoacán	273	0.70	0.00	0.70	0.70
Sierra de Santa Catarina I	Distrito Federal	525	0.70	0.00	0.70	0.70
Ing. Gerardo Cruickshank García	Estado de México	805	0.70	0.00	0.70	0.70
El Cedral	Zacatecas	996	0.70	0.00	0.70	0.70
Ejido San Juan de Guadalupe	San Luis Potosí	1194	0.70	0.00	0.70	0.70
Santuario del Agua Lagunas de Xico	Estado de México	1428	0.70	0.00	0.70	0.70
La Hediondilla	Nuevo León	4334	0.70	0.00	0.70	0.70
Chapa de Mota	Estado de México	5790	0.70	0.14	0.63	1.00
Parque Francisco Zarco	Michoacán	18	0.67	0.22	0.47	0.91
Cuenca Alta del Río Temascatío	Guanajuato	17278	0.67	0.20	0.41	0.92
Santuario del Agua Manantiales de Tiacaque	Estado de México	2181	0.66	0.05	0.63	0.76
Llano de la Soledad	Nuevo León	7526	0.66	0.09	0.48	0.76
Volcán Tacaná	Chiapas	4345	0.64	0.18	0.15	0.91
Huiricuta y la Ruta Histórica Cultural del Pueblo Huichol	San Luis Potosí	138751	0.64	0.14	0.41	0.92
Laguna de Yuriria y su Zona de Influencia	Guanajuato	15010	0.64	0.23	0.41	0.91
Bosque El Hiloche (pol 2)	Hidalgo	0	0.63	0.00	0.63	0.63
Bosque El Hiloche (pol 3)	Hidalgo	2	0.63	0.00	0.63	0.63
El Ocotal	Estado de México	166	0.63	0.00	0.63	0.63
Isla de las Aves	Estado de México	194	0.63	0.00	0.63	0.63
El Llano	Estado de México	240	0.63	0.00	0.63	0.63
Parque Ecológico El Tecuán	Durango	885	0.63	0.00	0.63	0.63
Bosque El Hiloche (pol 4)	Hidalgo	44	0.63	0.00	0.63	0.63
Bosque El Hiloche (pol 1)	Hidalgo	54	0.63	0.00	0.63	0.63

Pico El Loro-Paxtal	Chiapas	61554	0.63	0.22	0.15	0.91
Cerro del Muerto	Aguascalientes	5798	0.62	0.13	0.47	0.91
Loma de Santa María y Depresiones Aledañas	Michoacán	231	0.62	0.21	0.47	0.91
Cerro del Cubilete	Guanajuato	3574	0.61	0.23	0.05	0.91
Cerros El Culiacán y La Gavia	Guanajuato	32402	0.60	0.21	0.41	0.91
Parque Ecológico Agua Tibia-Jeroche	Michoacán	682	0.52	0.15	0.47	0.91
La Quemada	Zacatecas	165	0.52	0.08	0.47	0.70
Lago Cráter La Joya	Guanajuato	1441	0.52	0.18	0.41	0.91
Sierra de San Juan	Nayarit	19628	0.49	0.34	0.05	0.91
Predio Barragán	Veracruz	2	0.49	0.00	0.49	0.49
Pacho Nuevo	Veracruz	3	0.49	0.00	0.49	0.49
Cerro de las Culebras	Veracruz	35	0.49	0.00	0.49	0.49
El Tejar Garnica	Veracruz	92	0.49	0.00	0.49	0.49
Laguna de Zacapu y su Rivera	Michoacán	54	0.48	0.16	0.47	0.70
La Alberca de Los Espinos	Michoacán	141	0.47	0.00	0.47	0.47
Manantial La Mintzita y su Zona de Amortiguamiento	Michoacán	415	0.47	0.00	0.47	0.47
Instituto Tec. Agropecuario No 7	Michoacán	7	0.44	0.03	0.41	0.47
Presa Neutla y su Zona de Influencia	Guanajuato	1993	0.43	0.03	0.41	0.47
Hierve El Agua	Oaxaca	4129	0.42	0.29	0.05	0.91
San Juan y Puentes	Nuevo León	18	0.41	0.00	0.41	0.41
Bordo Benito Juárez	Querétaro	27	0.41	0.00	0.41	0.41
Ecológico del Fideicomiso de la Cd. Industrial de Morelia	Michoacán	88	0.41	0.00	0.41	0.41
Las Fuentes	Guanajuato	108	0.41	0.00	0.41	0.41
La Trinidad (2000)	Nuevo León	131	0.41	0.00	0.41	0.41
El Refugio de Apanaco	Nuevo León	823	0.41	0.00	0.41	0.41
Acuña	Nuevo León	1217	0.41	0.00	0.41	0.41
Trinidad y Llano Salas	Nuevo León	1988	0.41	0.00	0.41	0.41
Las Musas	Guanajuato	3164	0.41	0.00	0.41	0.41
Real de Guadalcázar	San Luis Potosí	254617	0.40	0.36	0.05	0.92
El Gancho Murillo	Chiapas	6722	0.39	0.40	0.11	1.00
El Cielo	Tamaulipas	135038	0.38	0.34	0.05	0.92
Arivechi Cerro Las Conchas	Sonora	67862	0.36	0.17	0.05	0.54
Parque Metropolitano	Guanajuato	337	0.35	0.14	0.05	0.41
Sierra de Otontepec	Veracruz	14429	0.34	0.20	0.15	0.65
Siete Luminarias	Guanajuato	8802	0.33	0.26	0.05	0.91
Sierra Las Mitras	Nuevo León	3326	0.30	0.38	0.05	0.91
Presa de Silva	Guanajuato	8701	0.30	0.17	0.05	0.41
Sierra El Fraile y San Miguel	Nuevo León	23281	0.30	0.35	0.05	0.91
Río Filobobos y su Entorno	Veracruz	10453	0.27	0.21	0.15	0.65
Presa La Purísima y su Zona de Influencia	Guanajuato	2696	0.25	0.18	0.05	0.41
El Cabildo Amatal	Chiapas	4017	0.25	0.32	0.11	1.00
Barranca de Chapultepec	Morelos	20	0.21	0.00	0.21	0.21

Cerro del Fortín	Oaxaca	87	0.21	0.00	0.21	0.21
Media Luna	San Luis Potosí	282	0.21	0.00	0.21	0.21
Sierra de Nanchititla	Estado de México	65026	0.20	0.24	0.00	0.91
Sierra Corral de los Bandidos	Nuevo León	1156	0.19	0.18	0.05	0.41
El Texcal	Morelia	414	0.19	0.03	0.15	0.21
Cerro El Topo	Nuevo León	1091	0.19	0.06	0.05	0.21
Tatocapan	Veracruz	1	0.19	0.00	0.19	0.19
El Bosque Adolfo Roque Bautista	San Luis Potosí	30	0.19	0.00	0.19	0.19
Playa Verde Camacho (1)	Sinaloa	67	0.19	0.00	0.19	0.19
Santuario del Loro Huasteco	Veracruz	68	0.19	0.00	0.19	0.19
Manantiales de Parácuaro	Michoacán	70	0.19	0.00	0.19	0.19
Sierra del Este y Sierra de Enmedio	San Luis Potosí	1777	0.19	0.00	0.19	0.19
Ciénega del Fuerte	Veracruz	4235	0.19	0.00	0.19	0.19
La Concordia Zaragoza	Chiapas	16525	0.18	0.06	0.15	0.49
Parque Lineal (Río Santa Catarina)	Nuevo León	688	0.17	0.03	0.15	0.21
Sierra de Montenegro	Morelia	7296	0.17	0.03	0.05	0.21
Navachiste	Sinaloa	16887	0.17	0.05	0.00	0.19
La Pera	Chiapas	7497	0.17	0.02	0.15	0.21
El Zapotal	Chiapas	191	0.17	0.02	0.15	0.19
Sierra de Vallejo	Nayarit	63094	0.17	0.07	0.00	0.65
La Sepultura	Chiapas	11852	0.16	0.03	0.06	0.19
Malpais de Santo Tomás de los Plátanos	Estado de México	144	0.16	0.08	0.05	0.21
Tehuacán - Zapotitlán	Puebla	2844	0.16	0.02	0.15	0.21
El Mineral de Nuestra Señora de la Candelaria	Sinaloa	1218	0.16	0.02	0.15	0.19
Las Cuevas del Viento y la Fertilidad	San Luis Potosí	8	0.16	0.03	0.15	0.21
La Chichihua	Michoacán	39	0.16	0.03	0.15	0.21
Altas Cumbres	Tamaulipas	31251	0.16	0.07	0.05	0.23
El Barrancón de las Guacamayas	Michoacán	2251	0.16	0.04	0.05	0.21
Las Estacas	Morelia	650	0.16	0.02	0.15	0.19
El Sótano de Las Golondrinas	San Luis Potosí	280	0.15	0.07	0.05	0.21
Cascadas de Cuatenáhuatl	Hidalgo	18	0.15	0.00	0.15	0.15
Parque Público Cerro del Obispado	Nuevo León	18	0.15	0.00	0.15	0.15
Palma Larga	San Luis Potosí	25	0.15	0.00	0.15	0.15
Los Chorros del Varal	Michoacán	72	0.15	0.00	0.15	0.15
La Lluvia	Chiapas	108	0.15	0.00	0.15	0.15
Los Sabinos - Santa Rosa - San Cristóbal (Río Cuautla)	Morelia	186	0.15	0.00	0.15	0.15
La Hoya de las Huahuas	San Luis Potosí	403	0.15	0.00	0.15	0.15
Volcán El Jorullo	Michoacán	3549	0.15	0.01	0.15	0.19
Cerro Meyapac	Chiapas	1748	0.15	0.00	0.15	0.15
San Elías	Nuevo León	655	0.15	0.17	0.05	0.41
Baño de San Ignacio	Nuevo León	4194	0.15	0.00	0.15	0.15
Parras de la Fuente	Tamaulipas	21727	0.15	0.00	0.15	0.15
Sistema de Presas Abelardo Rodríguez Luján - El Molinito	Sonora	28088	0.15	0.00	0.15	0.15

Las Flores	Nuevo León	81	0.15	0.00	0.15	0.15
Laguna La Escondida	Tamaulipas	318	0.15	0.00	0.15	0.15
Estero San José del Cabo	Baja California Sur	468	0.15	0.00	0.15	0.15
Cerro Mactumatza	Chiapas	611	0.15	0.00	0.15	0.15
Vaquerías	Nuevo León	1110	0.15	0.00	0.15	0.15
Sierra Picachos	Nuevo León	75232	0.13	0.09	0.05	0.86
Parque Ecológico Regional del Itsmo	Oaxaca	183	0.11	0.00	0.11	0.11
La Encrucijada	Chiapas	8884	0.11	0.00	0.11	0.11
Sierra Cerro de la Silla	Nuevo León	10512	0.11	0.08	0.05	0.23
Cañón de Fernández	Durango	16857	0.11	0.08	0.05	0.21
Río Grande San Pedro	Estado de México	87021	0.11	0.07	0.00	0.21
Refugio Estatal de Flora y Fauna Sistema Lagunar Chacmochuch	Quintana Roo	1897	0.07	0.21	0.00	1.00
Sierra de Tabasco	Tabasco	16746	0.07	0.08	0.00	0.19
Cerro de Arandas	Guanajuato	5205	0.06	0.08	0.05	0.41
Santuario del Manatí, Bahía de Chetumal	Quintana Roo	276455	0.06	0.08	0.06	1.00
Humedales La Libertad	Chiapas	5431	0.06	0.01	0.06	0.11
Reserva El Palmar	Yucatán	49051	0.06	0.04	0.06	1.00
Sierra de Huautla	Morelia	1396	0.06	0.08	0.00	0.19
La Laguna de Chandio	Michoacán	12	0.06	0.00	0.06	0.06
Laguna de Chancanaab	Quintana Roo	14	0.06	0.00	0.06	0.06
Parque Ecológico Laguna La Lima	Tabasco	22	0.06	0.00	0.06	0.06
Kabah	Quintana Roo	41	0.06	0.00	0.06	0.06
Laguna del Camarón	Tabasco	51	0.06	0.00	0.06	0.06
Laguna de Manatí	Quintana Roo	201	0.06	0.00	0.06	0.06
Laguna de las Ilusiones	Tabasco	222	0.06	0.00	0.06	0.06
Parque Ecológico de la Chontalpa	Tabasco	305	0.06	0.00	0.06	0.06
Fracción del Predio Santa Ana	Chiapas	512	0.06	0.00	0.06	0.06
Yu - Balcah	Tabasco	558	0.06	0.00	0.06	0.06
Río Playa	Tabasco	709	0.06	0.00	0.06	0.06
Lagunas Costeras y Serranías Aledañas de la Costa de Michoacán (pol 1)	Michoacán	1068	0.06	0.00	0.06	0.06
Refugio Estatal de Flora y Fauna Laguna Colombia	Quintana Roo	1502	0.06	0.00	0.06	0.06
Centro de Interpretación de la Naturaleza (Yumka)	Tabasco	1516	0.06	0.00	0.06	0.06
Cascadas de Reforma	Tabasco	5583	0.06	0.00	0.06	0.06
Sistema Lagunar Catazaja	Chiapas	41033	0.06	0.00	0.06	0.06
Los Petenes	Campeche	68194	0.06	0.00	0.06	0.06
Lagunas Costeras y Serranías Aledañas de la Costa de Michoacán (pol 2)	Michoacán	3223	0.05	0.07	0.00	0.19
Agua Caliente	Michoacán	38	0.05	0.21	0.05	0.41
Bernal de Horcasitas o Cerro de Bernal	Tamaulipas	18019	0.05	0.08	0.00	0.19
Grutas de la Estrella	Estado de México	4	0.05	0.00	0.05	0.05
Ecológico de Taquiscuareo	Michoacán	12	0.05	0.00	0.05	0.05
Bosque Cuauhtemoc	Michoacán	22	0.05	0.00	0.05	0.05
Parque Juárez	Michoacán	28	0.05	0.00	0.05	0.05

Cerro Hueco	Michoacán	33	0.05	0.00	0.05	0.05
La Alberca y su Zona de Influencia	Michoacán	44	0.05	0.00	0.05	0.05
La Eucalera de Paso de Hidalgo	Michoacán	76	0.05	0.00	0.05	0.05
Cerro La Mota	Nuevo León	9335	0.05	0.00	0.05	0.05
Gruta del Cerro Coconá	Tabasco	285	0.04	0.02	0.00	0.06
Balam-Kin	Campeche	96851	0.03	0.05	0.00	0.19
Balam-Ku	Campeche	406960	0.01	0.02	0.00	0.06
Reserva de Dzilam	Yucatán	68367	0.01	0.07	0.00	1.00
Medano del Perro	Veracruz	5	0.00	0.00	0.00	0.00
Isla del Amor (Punta Canales)	Veracruz	7	0.00	0.00	0.00	0.00
Santuario de la Tortuga Marina Xcacel - Xcacelito (Terrestre)	Quintana Roo	18	0.00	0.00	0.00	0.00
Tancojol	San Luis Potosí	95	0.00	0.00	0.00	0.00
El Canelar	Chiapas	110	0.00	0.00	0.00	0.00
Estero El Salado	Jalisco	210	0.00	0.00	0.00	0.00
Arroyo Moreno	Veracruz	291	0.00	0.00	0.00	0.00
Estero El Soldado	Sonora	313	0.00	0.00	0.00	0.00
Santuario de la Tortuga Marina Xcacel - Xcacelito (Marino)	Quintana Roo	343	0.00	0.00	0.00	0.00
Kabah	Yucatán	996	0.00	0.00	0.00	0.00
San Juan Bautista Tabi y Anexa Zac Nicte	Yucatán	1420	0.00	0.00	0.00	0.00
Agua Blanca	Tabasco	1879	0.00	0.00	0.00	0.00
La Sabana	Oaxaca	2048	0.00	0.00	0.00	0.00
Lagunas de Yalahau	Yucatán	5420	0.00	0.00	0.00	0.00

Table S4. Ecophysiological suitability index for *Batrachochytrium dendrobatidis* in the distribution of 273 amphibian species endemic to Mexico. Red list category, population trend and habitat are based on data available from IUCN.

Species	Redlist Category	Population Trend	Family	Habitat	mean	σ	min	max
Ambystoma leorae	Critically Endangered	Decreasing	Ambystomatidae	Terrestrial/Freshwater	1.00	0.00	1.00	1.00
Chiropterotriton ceronorum	Critically Endangered	Decreasing	Plethodontidae	Terrestrial	1.00	0.00	1.00	1.00
Chiropterotriton perotensis	Critically Endangered	Decreasing	Plethodontidae	Terrestrial	1.00	0.00	1.00	1.00
Pseudoeurycea robertsi	Critically Endangered	Decreasing	Plethodontidae	Terrestrial	1.00	0.00	1.00	1.00
Ambystoma rivulare	Endangered	Decreasing	Ambystomatidae	Freshwater	0.99	0.05	0.63	1.00
Pseudoeurycea aurantia	Critically Endangered	Decreasing	Plethodontidae	Terrestrial	0.98	0.07	0.63	1.00
Thorius spilogaster	Critically Endangered	Decreasing	Plethodontidae	Terrestrial	0.96	0.11	0.63	1.00
Pseudoeurycea papenfussi	Endangered	Decreasing	Plethodontidae	Terrestrial	0.96	0.11	0.63	1.00
Thorius lunaris	Critically Endangered	Decreasing	Plethodontidae	Terrestrial	0.95	0.12	0.63	1.00
Thorius aureus	Critically Endangered	Decreasing	Plethodontidae	Terrestrial	0.92	0.14	0.63	1.00
Ambystoma altamirani	Endangered	Decreasing	Ambystomatidae	Terrestrial/Freshwater	0.91	0.15	0.63	1.00
Pseudoeurycea gadovii	Vulnerable	Decreasing	Plethodontidae	Terrestrial	0.90	0.15	0.49	1.00
Dendrotriton megarhinus	Vulnerable	Unknown	Plethodontidae	Terrestrial	0.89	0.03	0.86	0.91
Thorius papaloae	Critically Endangered	Decreasing	Plethodontidae	Terrestrial	0.89	0.14	0.63	1.00
Chiropterotriton arboreus	Critically Endangered	Decreasing	Plethodontidae	Terrestrial	0.88	0.05	0.70	0.91
Chiropterotriton aureus	Critically Endangered	Unknown	Plethodontidae	Terrestrial	0.88	0.03	0.86	0.91
Pseudoeurycea melanomolga	Endangered	Decreasing	Plethodontidae	Terrestrial	0.87	0.14	0.49	1.00
Isthmura naucampatepetl	Critically Endangered	Decreasing	Plethodontidae	Terrestrial	0.87	0.14	0.63	1.00

Pseudoeurycea firscheini	Endangered	Decreasing	Plethodontidae	Terrestrial	0.86	0.16	0.63	1.00
Sarcohyla miahuatlanensis	Data Deficient	Unknown	Hylidae	Terrestrial/Freshwater	0.85	0.10	0.70	0.91
Thorius troglodytes	Endangered	Decreasing	Plethodontidae	Terrestrial	0.84	0.16	0.63	1.00
Pseudoeurycea smithi	Critically Endangered	Decreasing	Plethodontidae	Terrestrial	0.84	0.16	0.63	1.00
Thorius narisovalis	Endangered	Decreasing	Plethodontidae	Terrestrial	0.84	0.15	0.63	1.00
Thorius magnipes	Critically Endangered	Decreasing	Plethodontidae	Terrestrial	0.84	0.16	0.63	1.00
Sarcohyla calvicollina	Critically Endangered	Decreasing	Hylidae	Terrestrial/Freshwater	0.82	0.14	0.63	1.00
Thorius schmidti	Critically Endangered	Decreasing	Plethodontidae	Terrestrial	0.82	0.17	0.63	1.00
Chiropterotriton orculus	Vulnerable	Decreasing	Plethodontidae	Terrestrial	0.82	0.15	0.05	1.00
Incilius mccoyi	Least Concern	Stable	Bufonidae	Terrestrial/Freshwater	0.82	0.19	0.05	1.00
Chiropterotriton chico	Vulnerable	Stable	Plethodontidae	Terrestrial	0.81	0.18	0.63	1.00
Chiropterotriton dimidiatus	Vulnerable	Stable	Plethodontidae	Terrestrial	0.81	0.18	0.63	1.00
Pseudoeurycea leprosa	Least Concern	Decreasing	Plethodontidae	Terrestrial	0.81	0.16	0.05	1.00
Thorius boreas	Endangered	Decreasing	Plethodontidae	Terrestrial	0.81	0.17	0.05	1.00
Pseudoeurycea longicauda	Endangered	Decreasing	Plethodontidae	Terrestrial	0.81	0.16	0.63	1.00
Pseudoeurycea lynchi	Endangered	Decreasing	Plethodontidae	Terrestrial	0.80	0.15	0.49	1.00
Chiropterotriton terrestris	Critically Endangered	Unknown	Plethodontidae	Terrestrial	0.80	0.10	0.49	0.91
Incilius spiculatus	Endangered	Decreasing	Bufonidae	Terrestrial/Freshwater	0.80	0.15	0.49	1.00
Chiropterotriton nubilus	Critically Endangered	Unknown	Plethodontidae	Terrestrial	0.80	0.08	0.70	0.91
Sarcohyla labedactyla	Critically Endangered	Decreasing	Hylidae	Terrestrial/Freshwater	0.80	0.10	0.49	0.91
Pseudoeurycea tlilicxitl	Endangered	Unknown	Plethodontidae	Terrestrial	0.80	0.13	0.63	1.00

Thorius dubitus	Critically Endangered	Decreasing	Plethodontidae	Terrestrial	0.79	0.17	0.63	1.00
Thorius grandis	Critically Endangered	Stable	Plethodontidae	Terrestrial	0.79	0.08	0.70	0.91
Thorius macdougalli	Endangered	Decreasing	Plethodontidae	Terrestrial	0.79	0.13	0.63	1.00
Pseudoeurycea mixcoatl	Critically Endangered	Decreasing	Plethodontidae	Terrestrial	0.78	0.14	0.49	0.91
Pseudoeurycea saltator	Critically Endangered	Decreasing	Plethodontidae	Terrestrial	0.78	0.09	0.70	0.91
Pseudoeurycea juarezi	Endangered	Decreasing	Plethodontidae	Terrestrial	0.78	0.21	0.15	1.00
Eleutherodactylus erendirae	Endangered	Unknown	Eleutherodactylidae	Terrestrial	0.78	0.20	0.05	0.91
Sarcohyla cembra	Endangered	Decreasing	Hylidae	Terrestrial/Freshwater	0.77	0.08	0.70	0.91
Eleutherodactylus manantlanensis	Critically Endangered	Unknown	Eleutherodactylidae	Terrestrial	0.77	0.06	0.70	0.91
Thorius arboreus	Critically Endangered	Decreasing	Plethodontidae	Terrestrial	0.77	0.10	0.63	1.00
Thorius pinicola	Endangered	Decreasing	Plethodontidae	Terrestrial	0.77	0.08	0.70	0.91
Craugastor taylori	Critically Endangered	Unknown	Craugastoridae	Terrestrial	0.77	0.14	0.49	0.91
Dryophytes plicatus	Least Concern	Stable	Hylidae	Terrestrial/Freshwater	0.77	0.14	0.05	1.00
Sarcohyla chryses	Endangered	Decreasing	Hylidae	Terrestrial/Freshwater	0.76	0.16	0.49	0.91
Craugastor glaucus	Endangered	Decreasing	Craugastoridae	Terrestrial	0.76	0.03	0.70	0.91
Chiropterotriton lavae	Critically Endangered	Decreasing	Plethodontidae	Terrestrial	0.76	0.06	0.63	0.91
Thorius hankeni	Data Deficient	Unknown	Plethodontidae	Terrestrial	0.76	0.09	0.70	0.91
Thorius longicaudus	Critically Endangered	Unknown	Plethodontidae	Terrestrial	0.76	0.09	0.49	0.91
Pseudoeurycea altamontana	Endangered	Decreasing	Plethodontidae	Terrestrial	0.76	0.15	0.63	1.00
Ambystoma taylori	Critically Endangered	Unknown	Ambystomatidae	Freshwater	0.76	0.00	0.76	0.76
Chiropterotriton totonacus	Critically Endangered	Decreasing	Plethodontidae	Terrestrial	0.76	0.00	0.76	0.76

Lithobates chichicuahutla	Critically Endangered	Decreasing	Ranidae	Terrestrial/Freshwater	0.76	0.00	0.76	0.76
Thorius minutissimus	Critically Endangered	Decreasing	Plethodontidae	Terrestrial	0.76	0.16	0.49	0.91
Thorius pulmonaris	Critically Endangered	Decreasing	Plethodontidae	Terrestrial	0.76	0.12	0.63	1.00
Eleutherodactylus grandis	Endangered	Decreasing	Eleutherodactylidae	Terrestrial	0.76	0.01	0.70	0.76
Pseudoeurycea teotepec	Critically Endangered	Decreasing	Plethodontidae	Terrestrial	0.76	0.02	0.63	0.76
Pseudoeurycea mystax	Endangered	Decreasing	Plethodontidae	Terrestrial	0.76	0.12	0.49	0.91
Chiropterotriton chondrostega	Endangered	Decreasing	Plethodontidae	Terrestrial	0.76	0.17	0.05	0.92
Sarcohyla crassa	Critically Endangered	Decreasing	Hylidae	Terrestrial/Freshwater	0.75	0.21	0.05	1.00
Ambystoma lermaense	Endangered	Decreasing	Ambystomatidae	Terrestrial/Freshwater	0.75	0.03	0.63	1.00
Sarcohyla ameibothalame	Endangered	Decreasing	Hylidae	Terrestrial/Freshwater	0.75	0.04	0.63	0.91
Eleutherodactylus floresvillelai	Vulnerable	Unknown	Eleutherodactylidae	Terrestrial	0.75	0.02	0.70	0.76
Ambystoma amblycephalum	Critically Endangered	Decreasing	Ambystomatidae	Terrestrial/Freshwater	0.75	0.02	0.70	0.76
Chiropterotriton miquihuanus	Endangered	Stable	Plethodontidae	Terrestrial	0.75	0.03	0.63	0.91
Thorius minydemus	Endangered	Decreasing	Plethodontidae	Terrestrial	0.75	0.09	0.63	1.00
Sarcohyla arborescandens	Near Threatened	Decreasing	Hylidae	Terrestrial/Freshwater	0.75	0.19	0.05	1.00
Pseudoeurycea obesa	Critically Endangered	Unknown	Plethodontidae	Terrestrial	0.75	0.10	0.63	1.00
Sarcohyla hazelae	Vulnerable	Decreasing	Hylidae	Terrestrial/Freshwater	0.75	0.18	0.05	1.00
Thorius tlaxiacus	Endangered	Decreasing	Plethodontidae	Terrestrial	0.75	0.06	0.63	1.00
Isthmura corrugata	Critically Endangered	Unknown	Plethodontidae	Terrestrial	0.74	0.02	0.70	0.76
Eleutherodactylus wixarika	Endangered	Decreasing	Eleutherodactylidae	Terrestrial	0.74	0.05	0.63	0.76
Thorius maxillabrochus	Endangered	Decreasing	Plethodontidae	Terrestrial	0.74	0.13	0.63	1.00

Isthmura gigantea	Endangered	Decreasing	Plethodontidae	Terrestrial	0.73	0.20	0.05	1.00
Ambystoma granulosum	Endangered	Decreasing	Ambystomatidae	Terrestrial/Freshwater	0.73	0.10	0.63	1.00
Ambystoma mexicanum	Critically Endangered	Decreasing	Ambystomatidae	Freshwater	0.73	0.07	0.63	1.00
Chiropterotriton mosaueri	Critically Endangered	Unknown	Plethodontidae	Terrestrial	0.72	0.06	0.70	0.91
Lithobates dunni	Endangered	Decreasing	Ranidae	Freshwater	0.72	0.17	0.05	0.91
Chiropterotriton chiropterus	Critically Endangered	Decreasing	Plethodontidae	Terrestrial	0.72	0.17	0.49	1.00
Pseudoeurycea tenchalli	Critically Endangered	Decreasing	Plethodontidae	Terrestrial	0.71	0.19	0.49	0.91
Pseudoeurycea cochranae	Vulnerable	Decreasing	Plethodontidae	Terrestrial	0.71	0.20	0.05	1.00
Ambystoma silvense	Data Deficient	Unknown	Ambystomatidae	Terrestrial/Freshwater	0.71	0.13	0.54	1.00
Chiropterotriton infernalis	Least Concern	Stable	Plethodontidae	Terrestrial	0.71	0.29	0.05	0.91
Craugastor galacticorhinus	Endangered	Unknown	Craugastoridae	Terrestrial	0.71	0.20	0.05	1.00
Sarcohyla floresi	Vulnerable	Unknown	Hylidae	Terrestrial/Freshwater	0.70	0.18	0.05	0.92
Sarcohyla celata	Near Threatened	Stable	Hylidae	Terrestrial/Freshwater	0.70	0.23	0.05	1.00
Ambystoma dumerilii	Critically Endangered	Decreasing	Ambystomatidae	Freshwater	0.70	0.00	0.70	0.70
Pseudoeurycea ahuitzotl	Critically Endangered	Decreasing	Plethodontidae	Terrestrial	0.70	0.17	0.49	0.91
Bolitoglossa hartwegi	Vulnerable	Decreasing	Plethodontidae	Terrestrial	0.70	0.19	0.00	0.92
Aquiloeurycea galeanae	Vulnerable	Decreasing	Plethodontidae	Terrestrial	0.70	0.18	0.05	0.91
Thorius munificus	Critically Endangered	Decreasing	Plethodontidae	Terrestrial	0.70	0.11	0.63	1.00
Eleutherodactylus grunwaldi	Endangered	Stable	Eleutherodactylidae	Terrestrial	0.69	0.22	0.05	0.91
Ambystoma ordinarium	Endangered	Decreasing	Ambystomatidae	Terrestrial/Freshwater	0.69	0.20	0.05	1.00
Charadrahyla taeniopus	Vulnerable	Decreasing	Hylidae	Terrestrial/Freshwater	0.69	0.21	0.05	1.00

Megastomatohyla nubicola	Critically Endangered	Unknown	Hylidae	Terrestrial/Freshwater	0.69	0.18	0.49	0.86
Sarcohyla cyclada	Vulnerable	Decreasing	Hylidae	Terrestrial/Freshwater	0.69	0.26	0.05	1.00
Dendrotriton xolocalcae	Vulnerable	Stable	Plethodontidae	Terrestrial	0.68	0.22	0.15	0.91
Pseudoeurycea tlahcuiloh	Critically Endangered	Decreasing	Plethodontidae	Terrestrial	0.68	0.17	0.49	0.91
Incilius cristatus	Endangered	Decreasing	Bufonidae	Terrestrial/Freshwater	0.68	0.17	0.15	1.00
Cryptotriton alvarezdeltoroi	Endangered	Decreasing	Plethodontidae	Terrestrial	0.67	0.24	0.05	0.92
Chiropterotriton priscus	Near Threatened	Stable	Plethodontidae	Terrestrial	0.66	0.29	0.05	1.00
Sarcohyla thorectes	Endangered	Decreasing	Hylidae	Terrestrial/Freshwater	0.66	0.18	0.49	0.91
Eleutherodactylus maurus	Vulnerable	Unknown	Eleutherodactylidae	Terrestrial	0.65	0.30	0.05	1.00
Sarcohyla robertsorum	Vulnerable	Decreasing	Hylidae	Terrestrial/Freshwater	0.65	0.26	0.05	1.00
Thorius omiltemi	Endangered	Decreasing	Plethodontidae	Terrestrial	0.64	0.27	0.15	0.91
Sarcohyla toyota	Critically Endangered	Decreasing	Hylidae	Terrestrial/Freshwater	0.64	0.16	0.49	0.91
Pseudoeurycea mixteca	Vulnerable	Decreasing	Plethodontidae	Terrestrial	0.64	0.28	0.05	0.91
Craugastor batrachylus	Data Deficient	Unknown	Craugastoridae	Terrestrial	0.64	0.20	0.41	0.91
Aquiloeurycea quetzalanensis	Critically Endangered	Decreasing	Plethodontidae	Terrestrial	0.63	0.19	0.21	0.91
Aquiloeurycea cephalica	Least Concern	Decreasing	Plethodontidae	Terrestrial	0.63	0.30	0.05	1.00
Dryophytes euphorbiaceus	Least Concern	Stable	Hylidae	Terrestrial/Freshwater	0.63	0.29	0.05	1.00
Eleutherodactylus saxatilis	Near Threatened	Stable	Eleutherodactylidae	Terrestrial	0.62	0.31	0.05	1.00
Chiropterotriton multidentatus	Endangered	Stable	Plethodontidae	Terrestrial	0.61	0.28	0.05	0.86
Eleutherodactylus jaliscoensis	Endangered	Decreasing	Eleutherodactylidae	Terrestrial	0.61	0.30	0.05	0.92
Sarcohyla labeculata	Endangered	Unknown	Hylidae	Terrestrial/Freshwater	0.61	0.22	0.05	1.00
Sarcohyla mykter	Endangered	Decreasing	Hylidae	Terrestrial/Freshwater	0.61	0.26	0.05	0.92

Lithobates montezumae	Least Concern	Decreasing	Ranidae	Terrestrial/Freshwater	0.60	0.25	0.00	1.00
Aquiloeurycea cafetalera	Vulnerable	Stable	Plethodontidae	Terrestrial	0.60	0.27	0.05	1.00
Parvimolge townsendi	Vulnerable	Decreasing	Plethodontidae	Terrestrial	0.59	0.29	0.05	1.00
Pseudoeurycea ruficauda	Endangered	Decreasing	Plethodontidae	Terrestrial	0.59	0.21	0.15	0.76
Isthmura sierraoccidentalis	Vulnerable	Unknown	Plethodontidae	Terrestrial	0.59	0.13	0.41	0.76
Charadrahyla sakbah	Endangered	Unknown	Hylidae	Terrestrial/Freshwater	0.58	0.28	0.15	0.91
Thorius pennatulus	Endangered	Decreasing	Plethodontidae	Terrestrial	0.58	0.27	0.05	1.00
Isthmura boneti	Endangered	Decreasing	Plethodontidae	Terrestrial	0.56	0.31	0.05	1.00
Ambystoma velasci	Least Concern	Unknown	Ambystomatidae	Terrestrial/Freshwater	0.56	0.28	0.00	1.00
Lithobates spectabilis	Least Concern	Decreasing	Ranidae	Terrestrial/Freshwater	0.56	0.32	0.00	1.00
Exerodonta xera	Vulnerable	Unknown	Hylidae	Terrestrial/Freshwater	0.56	0.32	0.00	1.00
Eleutherodactylus verruculatus	Data Deficient	Unknown	Eleutherodactylidae	Terrestrial	0.56	0.14	0.49	0.86
Anaxyrus mexicanus	Least Concern	Decreasing	Bufonidae	Terrestrial/Freshwater	0.56	0.29	0.00	1.00
Chiropterotriton melipona	Endangered	Decreasing	Plethodontidae	Terrestrial	0.56	0.13	0.49	0.91
Megastomatohyla pellita	Critically Endangered	Decreasing	Hylidae	Terrestrial/Freshwater	0.55	0.11	0.15	0.86
Thorius adelos	Near Threatened	Unknown	Plethodontidae	Terrestrial	0.55	0.22	0.15	0.91
Eleutherodactylus guttilatus	Least Concern	Unknown	Eleutherodactylidae	Terrestrial/Freshwater	0.55	0.27	0.05	1.00
Megastomatohyla mixomaculata	Endangered	Decreasing	Hylidae	Terrestrial/Freshwater	0.55	0.29	0.05	0.92
Craugastor rhodopis	Least Concern	Stable	Craugastoridae	Terrestrial	0.54	0.32	0.05	1.00
Ixalotriton parvus	Critically Endangered	Decreasing	Plethodontidae	Terrestrial	0.54	0.13	0.49	0.86
Craugastor spatulatus	Endangered	Decreasing	Craugastoridae	Terrestrial	0.53	0.31	0.05	1.00

Ambystoma rosaceum	Least Concern	Unknown	Ambystomatidae	Terrestrial/Freshwater	0.53	0.30	0.00	1.00
Plectrohyla lacertosa	Endangered	Decreasing	Hylidae	Terrestrial/Freshwater	0.53	0.25	0.00	0.91
Eleutherodactylus angustidigitorum	Least Concern	Stable	Eleutherodactylidae	Terrestrial	0.52	0.31	0.00	1.00
Chiropterotriton magnipes	Endangered	Decreasing	Plethodontidae	Terrestrial	0.52	0.35	0.05	0.92
Pseudoeurycea kuautli	Critically Endangered	Decreasing	Plethodontidae	Terrestrial	0.51	0.06	0.49	0.65
Rheohyla miotympanum	Least Concern	Stable	Hylidae	Terrestrial/Freshwater	0.51	0.31	0.00	1.00
Thorius narismagnus	Critically Endangered	Decreasing	Plethodontidae	Terrestrial	0.51	0.20	0.15	0.86
Isthmura bellii	Least Concern	Unknown	Plethodontidae	Terrestrial	0.50	0.30	0.00	1.00
Charadrahyla esperancensis	Vulnerable	Unknown	Hylidae	Terrestrial/Freshwater	0.50	0.18	0.21	0.86
Charadrahyla tecuani	Data Deficient	Unknown	Hylidae	Terrestrial/Freshwater	0.50	0.20	0.15	0.65
Craugastor tarahumaraensis	Least Concern	Unknown	Craugastoridae	Terrestrial	0.50	0.31	0.00	1.00
Smilisca dentata	Endangered	Decreasing	Hylidae	Terrestrial/Freshwater	0.50	0.08	0.47	0.91
Charadrahyla nephila	Endangered	Unknown	Hylidae	Terrestrial/Freshwater	0.50	0.30	0.00	1.00
Craugastor pelorus	Vulnerable	Unknown	Craugastoridae	Terrestrial/Freshwater	0.49	0.33	0.00	0.91
Duellmanohyla chamulae	Endangered	Decreasing	Hylidae	Terrestrial/Freshwater	0.49	0.32	0.00	0.92
Craugastor mexicanus	Least Concern	Stable	Craugastoridae	Terrestrial	0.49	0.32	0.00	1.00
Pseudoeurycea conanti	Endangered	Unknown	Plethodontidae	Terrestrial	0.48	0.18	0.15	0.91
Anaxyrus compactilis	Least Concern	Unknown	Bufonidae	Terrestrial/Freshwater	0.48	0.28	0.00	1.00
Sarcohyla pentheter	Vulnerable	Stable	Hylidae	Terrestrial/Freshwater	0.48	0.28	0.00	0.92
Pseudoeurycea lineola	Endangered	Decreasing	Plethodontidae	Terrestrial	0.48	0.25	0.05	0.91
Bolitoglossa zapoteca	Endangered	Unknown	Plethodontidae	Terrestrial	0.48	0.32	0.15	0.91

Ptychohyla leonhardschultzei	Least Concern	Decreasing	Hylidae	Terrestrial/Freshwater	0.48	0.30	0.00	1.00
Dryophytes eximius	Least Concern	Stable	Hylidae	Terrestrial/Freshwater	0.47	0.32	0.00	1.00
Sarcohyla bistincta	Least Concern	Decreasing	Hylidae	Terrestrial/Freshwater	0.47	0.32	0.00	1.00
Ambystoma andersoni	Critically Endangered	Decreasing	Ambystomatidae	Freshwater	0.47	0.00	0.47	0.47
Incilius occidentalis	Least Concern	Stable	Bufonidae	Terrestrial/Freshwater	0.47	0.31	0.00	1.00
Pseudoeurycea werleri	Endangered	Unknown	Plethodontidae	Terrestrial	0.46	0.20	0.15	0.86
Exerodonta chimalapa	Endangered	Unknown	Hylidae	Terrestrial/Freshwater	0.46	0.21	0.15	0.86
Craugastor decoratus	Least Concern	Stable	Craugastoridae	Terrestrial	0.46	0.32	0.00	1.00
Craugastor uno	Vulnerable	Unknown	Craugastoridae	Terrestrial	0.45	0.29	0.00	0.91
Sarcohyla hapsa	Least Concern	Unknown	Hylidae	Terrestrial/Freshwater	0.45	0.33	0.00	1.00
Eleutherodactylus rufescens	Vulnerable	Decreasing	Eleutherodactylidae	Terrestrial	0.45	0.33	0.05	1.00
Eleutherodactylus dilatus	Least Concern	Stable	Eleutherodactylidae	Terrestrial	0.44	0.31	0.05	0.91
Tlalocohyla godmani	Vulnerable	Decreasing	Hylidae	Terrestrial/Freshwater	0.44	0.28	0.05	1.00
Lithobates neovolcanicus	Least Concern	Stable	Ranidae	Terrestrial/Freshwater	0.44	0.28	0.00	1.00
Chiropterotriton cracens	Vulnerable	Stable	Plethodontidae	Terrestrial	0.44	0.30	0.05	0.86
Charadrahyla altipotens	Endangered	Unknown	Hylidae	Terrestrial/Freshwater	0.43	0.20	0.05	0.86
Pseudoeurycea nigromaculata	Endangered	Decreasing	Plethodontidae	Terrestrial	0.42	0.23	0.05	0.91
Aquiloeurycea scandens	Least Concern	Stable	Plethodontidae	Terrestrial	0.42	0.34	0.05	0.92
Bolitoglossa chinanteca	Near Threatened	Stable	Plethodontidae	Terrestrial	0.41	0.28	0.15	1.00
Eleutherodactylus nietoi	Endangered	Unknown	Eleutherodactylidae	Terrestrial	0.40	0.28	0.05	0.92

Exerodonta melanomma	Vulnerable	Unknown	Hylidae	Terrestrial/Freshwater	0.40	0.30	0.00	1.00
Craugastor polymniae	Near Threatened	Unknown	Craugastoridae	Terrestrial	0.40	0.26	0.15	0.91
Duellmanohyla ignicolor	Near Threatened	Stable	Hylidae	Terrestrial/Freshwater	0.40	0.26	0.15	0.91
Eleutherodactylus verrucipes	Least Concern	Stable	Eleutherodactylidae	Terrestrial	0.40	0.33	0.05	1.00
Eleutherodactylus teretistes	Vulnerable	Unknown	Eleutherodactylidae	Terrestrial	0.39	0.33	0.00	0.92
Eleutherodactylus nitidus	Least Concern	Stable	Eleutherodactylidae	Terrestrial	0.39	0.32	0.00	1.00
Isthmura maxima	Endangered	Unknown	Plethodontidae	Terrestrial	0.38	0.28	0.05	0.91
Charadrahyla chaneque	Vulnerable	Decreasing	Hylidae	Terrestrial/Freshwater	0.38	0.25	0.05	0.91
Eleutherodactylus syristes	Least Concern	Stable	Eleutherodactylidae	Terrestrial	0.37	0.26	0.00	1.00
Craugastor berkenbuschii	Least Concern	Unknown	Craugastoridae	Terrestrial/Freshwater	0.37	0.31	0.00	1.00
Lithobates megapoda	Near Threatened	Decreasing	Ranidae	Terrestrial/Freshwater	0.36	0.31	0.00	1.00
Bolitoglossa macrinii	Endangered	Decreasing	Plethodontidae	Terrestrial	0.36	0.29	0.00	0.92
Megastomatohyla mixe	Critically Endangered	Unknown	Hylidae	Terrestrial/Freshwater	0.35	0.21	0.15	0.86
Lithobates psilonota	Least Concern	Unknown	Ranidae	Terrestrial/Freshwater	0.35	0.29	0.00	1.00
Thorius insperatus	Critically Endangered	Decreasing	Plethodontidae	Terrestrial	0.35	0.23	0.15	0.65
Eleutherodactylus longipes	Least Concern	Unknown	Eleutherodactylidae	Terrestrial	0.34	0.32	0.00	1.00
Craugastor omiltemanus	Least Concern	Stable	Craugastoridae	Terrestrial	0.34	0.27	0.00	0.92
Ambystoma flavipiperatum	Endangered	Decreasing	Ambystomatidae	Terrestrial/Freshwater	0.34	0.32	0.05	0.91
Chiropterotriton cieloensis	Vulnerable	Stable	Plethodontidae	Terrestrial	0.33	0.25	0.05	0.86
Pseudoeurycea orchimelas	Endangered	Decreasing	Plethodontidae	Terrestrial	0.32	0.22	0.00	0.86

Exerodonta bivocata	Endangered	Unknown	Hylidae	Terrestrial/Freshwater	0.32	0.21	0.00	0.91
Ixalotriton niger	Endangered	Decreasing	Plethodontidae	Terrestrial	0.32	0.17	0.15	0.86
Charadrahyla juanitae	Near Threatened	Unknown	Hylidae	Terrestrial/Freshwater	0.31	0.26	0.00	0.91
Lithobates lemosespinali	Data Deficient	Unknown	Ranidae	Terrestrial	0.31	0.21	0.05	0.54
Pseudoeurycea orchileucos	Endangered	Decreasing	Plethodontidae	Terrestrial	0.31	0.25	0.05	0.91
Eleutherodactylus cystignathoides	Least Concern	Stable	Eleutherodactylidae	Terrestrial	0.31	0.29	0.00	1.00
Craugastor megalotympanum	Endangered	Decreasing	Craugastoridae	Terrestrial	0.30	0.23	0.00	0.86
Lithobates zweifeli	Least Concern	Stable	Ranidae	Terrestrial/Freshwater	0.30	0.30	0.00	1.00
Bolitoglossa hermosa	Least Concern	Stable	Plethodontidae	Terrestrial	0.30	0.26	0.00	0.91
Lithobates magnaocularis	Least Concern	Unknown	Ranidae	Terrestrial/Freshwater	0.29	0.27	0.00	1.00
Exerodonta smaragdina	Least Concern	Decreasing	Hylidae	Terrestrial/Freshwater	0.29	0.29	0.00	1.00
Charadrahyla pinorum	Vulnerable	Unknown	Hylidae	Terrestrial/Freshwater	0.29	0.23	0.00	0.92
Thorius smithi	Critically Endangered	Decreasing	Plethodontidae	Terrestrial	0.28	0.17	0.15	0.65
Plectrohyla pycnochila	Critically Endangered	Unknown	Hylidae	Terrestrial/Freshwater	0.28	0.15	0.05	0.49
Lithobates pustulosus	Least Concern	Stable	Ranidae	Terrestrial/Freshwater	0.28	0.28	0.00	1.00
Lithobates sierramadrensis	Least Concern	Stable	Ranidae	Terrestrial/Freshwater	0.27	0.27	0.00	1.00
Craugastor occidentalis	Least Concern	Stable	Craugastoridae	Terrestrial	0.27	0.27	0.00	1.00
Thorius infernalis	Critically Endangered	Decreasing	Plethodontidae	Terrestrial	0.26	0.20	0.15	0.65
Incilius macrocristatus	Near Threatened	Decreasing	Bufonidae	Terrestrial/Freshwater	0.26	0.23	0.00	0.92
Craugastor montanus	Endangered	Decreasing	Craugastoridae	Terrestrial	0.26	0.26	0.00	0.91

Ptychohyla zophodes	Vulnerable	Decreasing	Hylidae	Terrestrial/Freshwater	0.25	0.22	0.00	0.91
Exerodonta sumichrasti	Least Concern	Decreasing	Hylidae	Terrestrial/Freshwater	0.25	0.25	0.00	1.00
Quilticohyla erythromma	Endangered	Decreasing	Hylidae	Terrestrial/Freshwater	0.25	0.19	0.15	0.65
Pseudoeurycea amuzga	Endangered	Decreasing	Plethodontidae	Terrestrial	0.25	0.20	0.13	0.86
Craugastor vocalis	Least Concern	Decreasing	Craugastoridae	Terrestrial	0.24	0.25	0.00	1.00
Craugastor saltator	Endangered	Unknown	Craugastoridae	Terrestrial/Freshwater	0.24	0.19	0.00	0.65
Dryophytes arboricola	Vulnerable	Unknown	Hylidae	Terrestrial/Freshwater	0.24	0.23	0.00	0.91
Tlalocohyla smithii	Least Concern	Stable	Hylidae	Terrestrial/Freshwater	0.23	0.26	0.00	1.00
Craugastor guerreroensis	Endangered	Decreasing	Craugastoridae	Terrestrial	0.22	0.15	0.00	0.65
Craugastor hobartsmithi	Least Concern	Stable	Craugastoridae	Terrestrial	0.22	0.26	0.00	1.00
Ecnomiohyla valancifer	Critically Endangered	Unknown	Hylidae	Terrestrial/Freshwater	0.21	0.15	0.13	0.82
Dermophis oaxacae	Least Concern	Stable	Dermophiidae	Terrestrial	0.21	0.24	0.00	1.00
Eleutherodactylus dennisi	Least Concern	Stable	Eleutherodactylidae	Terrestrial	0.21	0.14	0.00	0.91
Eleutherodactylus orarius	Least Concern	Stable	Eleutherodactylidae	Terrestrial	0.20	0.24	0.00	1.00
Lithobates johni	Vulnerable	Decreasing	Ranidae	Terrestrial/Freshwater	0.20	0.14	0.00	0.91
Lithobates omiltemanus	Endangered	Stable	Ranidae	Terrestrial/Freshwater	0.20	0.22	0.00	0.91
Bromeliohyla dendroscarta	Endangered	Decreasing	Hylidae	Terrestrial	0.20	0.11	0.00	0.86
Incilius cavifrons	Endangered	Decreasing	Bufonidae	Terrestrial/Freshwater	0.19	0.19	0.00	0.86
Eleutherodactylus modestus	Least Concern	Stable	Eleutherodactylidae	Terrestrial	0.19	0.23	0.00	1.00
Craugastor vulcani	Endangered	Decreasing	Craugastoridae	Terrestrial/Freshwater	0.19	0.20	0.00	1.00
Craugastor rugulosus	Least Concern	Unknown	Craugastoridae	Terrestrial/Freshwater	0.19	0.22	0.00	1.00

Dendropsophus sartori	Least Concern	Stable	Hylidae	Terrestrial/Freshwater	0.18	0.21	0.00	1.00
Exerodonta abdivita	Near Threatened	Unknown	Hylidae	Terrestrial/Freshwater	0.18	0.17	0.00	0.91
Incilius perplexus	Least Concern	Stable	Bufonidae	Terrestrial/Freshwater	0.18	0.25	0.00	1.00
Quilticohyla zoque	Endangered	Decreasing	Hylidae	Terrestrial/Freshwater	0.18	0.07	0.00	0.82
Craugastor pozo	Critically Endangered	Decreasing	Craugastoridae	Terrestrial	0.18	0.03	0.15	0.21
Incilius marmoreus	Least Concern	Stable	Bufonidae	Terrestrial/Freshwater	0.18	0.21	0.00	1.00
Quilticohyla acrochorda	Critically Endangered	Decreasing	Hylidae	Terrestrial/Freshwater	0.17	0.02	0.15	0.19
Charadrahyla trux	Endangered	Decreasing	Hylidae	Terrestrial/Freshwater	0.17	0.11	0.05	0.82
Eleutherodactylus interorbitalis	Least Concern	Stable	Eleutherodactylidae	Terrestrial	0.17	0.12	0.00	1.00
Agalychnis dacnicolor	Least Concern	Decreasing	Phyllomedusidae	Terrestrial/Freshwater	0.17	0.19	0.00	1.00
Incilius cycladen	Vulnerable	Decreasing	Bufonidae	Terrestrial/Freshwater	0.17	0.15	0.00	0.86
Bolitoglossa riletti	Endangered	Decreasing	Plethodontidae	Terrestrial	0.17	0.08	0.00	0.86
Bolitoglossa alberchi	Vulnerable	Decreasing	Plethodontidae	Terrestrial	0.16	0.15	0.00	0.86
Lithobates forreri	Least Concern	Stable	Ranidae	Terrestrial/Freshwater	0.15	0.15	0.00	1.00
Eleutherodactylus albolabris	Least Concern	Stable	Eleutherodactylidae	Terrestrial	0.15	0.17	0.00	0.91
Incilius gemmifer	Endangered	Stable	Bufonidae	Terrestrial/Freshwater	0.14	0.14	0.00	0.82
Bolitoglossa platydactyla	Least Concern	Stable	Plethodontidae	Terrestrial	0.14	0.19	0.00	1.00
Anaxyrus kelloggi	Least Concern	Stable	Bufonidae	Terrestrial/Freshwater	0.14	0.08	0.00	1.00
Eleutherodactylus pallidus	Least Concern	Stable	Eleutherodactylidae	Terrestrial	0.14	0.18	0.00	1.00
Triprion spatulatus	Least Concern	Stable	Hylidae	Terrestrial/Freshwater	0.14	0.17	0.00	1.00
Incilius mazatlanensis	Least Concern	Stable	Bufonidae	Terrestrial/Freshwater	0.12	0.11	0.00	1.00
Incilius pisinnus	Endangered	Decreasing	Bufonidae	Terrestrial/Freshwater	0.09	0.08	0.00	0.21

Eleutherodactylus colimotl	Least Concern	Stable	Eleutherodactylidae	Terrestrial	0.08	0.10	0.00	0.65
Bolitoglossa veracrucis	Endangered	Decreasing	Plethodontidae	Terrestrial	0.08	0.09	0.00	0.82
Craugastor silvicola	Data Deficient	Unknown	Craugastoridae	Terrestrial	0.03	0.03	0.00	0.06
Craugastor yucatanensis	Near Threatened	Decreasing	Craugastoridae	Terrestrial	0.01	0.02	0.00	1.00

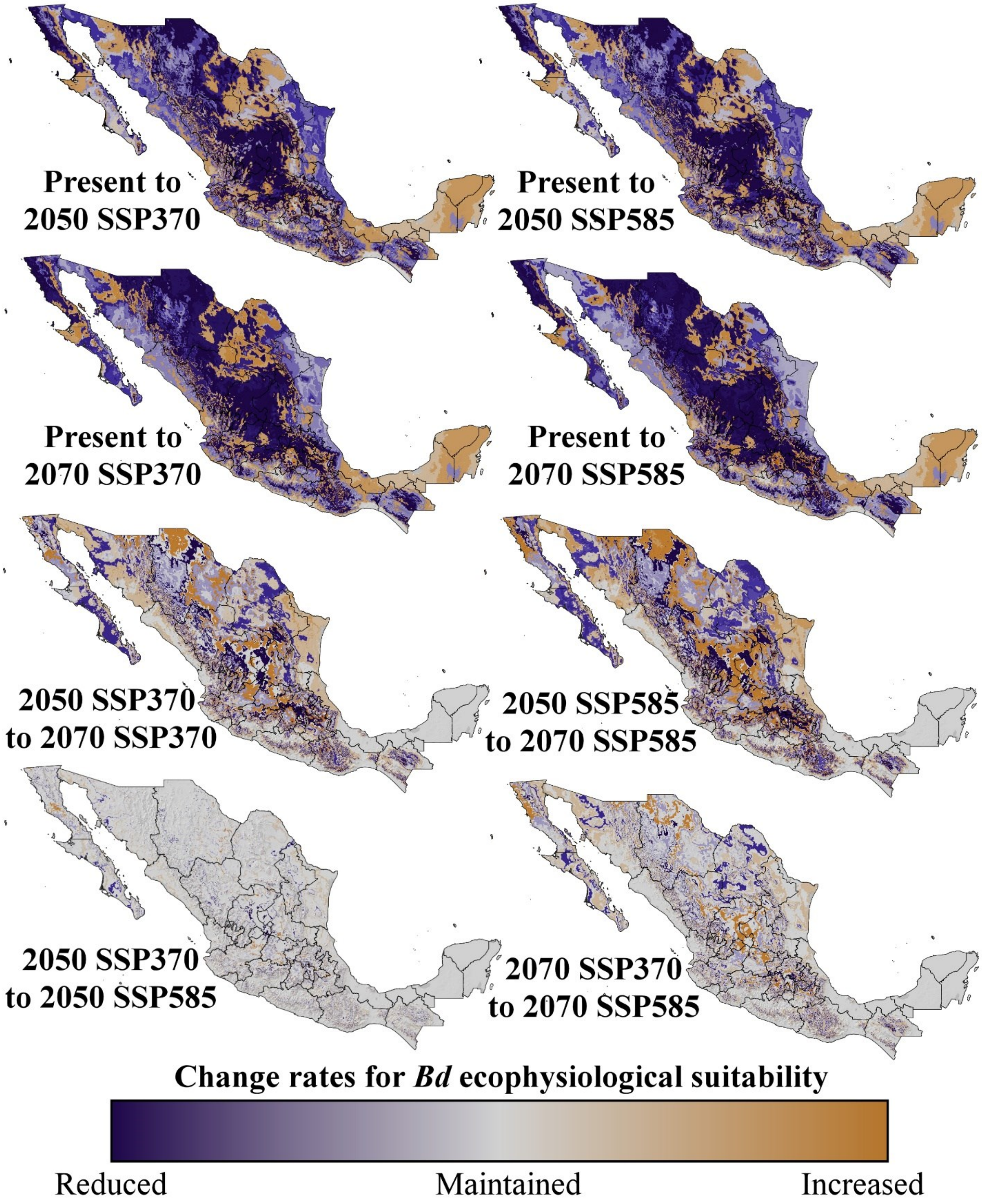

Figure S1. Change rates for *Batrachochytrium dendrobatidis* ecophysiological suitability between the present, 2050 and 2070 under two contrasting climate change scenarios, SSP370 and SSP585, according to ACCESS-CM2 General Circulation Model.
